# New World Bats Harbor Diverse Influenza A Viruses

**DOI:** 10.1371/journal.ppat.1003657

**Published:** 2013-10-10

**Authors:** Suxiang Tong, Xueyong Zhu, Yan Li, Mang Shi, Jing Zhang, Melissa Bourgeois, Hua Yang, Xianfeng Chen, Sergio Recuenco, Jorge Gomez, Li-Mei Chen, Adam Johnson, Ying Tao, Cyrille Dreyfus, Wenli Yu, Ryan McBride, Paul J. Carney, Amy T. Gilbert, Jessie Chang, Zhu Guo, Charles T. Davis, James C. Paulson, James Stevens, Charles E. Rupprecht, Edward C. Holmes, Ian A. Wilson, Ruben O. Donis

**Affiliations:** 1 Division of Viral Diseases, Centers for Disease Control and Prevention, Atlanta, Georgia, United States of America; 2 Department of Integrative Structural and Computational Biology, The Scripps Research Institute, La Jolla, California, United States of America; 3 Sydney Emerging Infections and Biosecurity Institute, School of Biological Sciences and Sydney Medical School, The University of Sydney, Sydney, New South Wales, Australia; 4 Influenza Division, Centers for Disease Control and Prevention, Atlanta, Georgia, United States of America; 5 Division of High Consequence Pathogens and Pathology, Centers for Disease Control and Prevention, Atlanta, Georgia, United States of America; 6 Direccion General de Epidemiologıa, Ministerio de Salud - MINSA, Lima, Peru; 7 Department of Chemical Physiology, The Scripps Research Institute, La Jolla, California, United States of America; 8 Global Alliance for Rabies Control, The Scripps Research Institute, La Jolla, California, United States of America; 9 Fogarty International Center, National Institutes of Health, Bethesda, Maryland, United States of America; 10 Skaggs Institute for Chemical Biology, The Scripps Research Institute, La Jolla, California, United States of America; National Institutes of Health, United States of America

## Abstract

Aquatic birds harbor diverse influenza A viruses and are a major viral reservoir in nature. The recent discovery of influenza viruses of a new H17N10 subtype in Central American fruit bats suggests that other New World species may similarly carry divergent influenza viruses. Using consensus degenerate RT-PCR, we identified a novel influenza A virus, designated as H18N11, in a flat-faced fruit bat (*Artibeus planirostris*) from Peru. Serologic studies with the recombinant H18 protein indicated that several Peruvian bat species were infected by this virus. Phylogenetic analyses demonstrate that, in some gene segments, New World bats harbor more influenza virus genetic diversity than all other mammalian and avian species combined, indicative of a long-standing host-virus association. Structural and functional analyses of the hemagglutinin and neuraminidase indicate that sialic acid is not a ligand for virus attachment nor a substrate for release, suggesting a unique mode of influenza A virus attachment and activation of membrane fusion for entry into host cells. Taken together, these findings indicate that bats constitute a potentially important and likely ancient reservoir for a diverse pool of influenza viruses.

## Author Summary

Previous studies indicated that a novel influenza A virus (H17N10) was circulating in fruit bats from Guatemala (Central America). Herein, we investigated whether similar viruses are present in bat species from South America. Analysis of rectal swabs from bats sampled in the Amazon rainforest region of Peru identified another new influenza A virus from bats that is phylogenetically distinct from the one identified in Guatemala. The genes that encode the surface proteins of the new virus from the flat-faced fruit bat were designated as new subtype H18N11. Serologic testing of blood samples from several species of Peruvian bats indicated a high prevalence of antibodies to the surface proteins. Phylogenetic analyses demonstrate that bat populations from Central and South America maintain as much influenza virus genetic diversity in some gene segments as all other mammalian and avian species combined. The crystal structures of the hemagglutinin and neuraminidase proteins indicate that sialic acid is not a receptor for virus attachment nor a substrate for release, suggesting a novel mechanism of influenza A virus attachment and activation of membrane fusion for entry into host cells. In summary, our findings indicate that bats constitute a potentially important reservoir for influenza viruses.

## Introduction

Influenza viruses originating from animals caused the three major influenza pandemics of the previous century. Swine were a gateway for avian influenza virus genes to enter human populations as reassortant viruses and also initiated the influenza (H1N1) pandemic of 2009 [1]. The recent discovery of novel influenza A viruses in Guatemalan yellow-shouldered fruit bats identified another mammalian species that may serve as an important source of influenza virus genetic diversity and support reassortment with human influenza viruses [2]. Bats are a major source of emerging infectious diseases, including coronaviruses, filoviruses, henipaviruses, and lyssaviruses [3]–[5]. Their global distribution, abundance, diversity (∼1200 species) and high population densities underscore the need to better understand the ecology and properties of influenza viruses that infect this mammalian order, as well as the potential of viral jumps across species barriers to emerge in new hosts [5]. These considerations prompted additional searches for species harboring novel influenza A viruses within the Americas, and detailed characterization of the virus-host interactions at the molecular and atomic levels.

## Results

### A novel influenza virus in Peruvian bats

We sampled 114 Peruvian bats captured during 2010 in Truenococha and Santa Marta, two communities located in the Loreto Department, Peru, a remote and sparsely populated area in Amazonia (Table 1 and Fig. S1). The sampled bats comprised 18 species, although 12 species were represented by four or fewer animals (Table 1). Initial screening of the available 110 rectal swabs with a pan Flu RT-PCR assay identified a flat-faced fruit bat (ID PEBT033) (*Artibeus planirostris*) from Truenococha as positive for influenza virus. Of the other available specimens, i.e. liver, intestine and spleen tissues from bat PEBT033, the intestine tissue specimen was strong positive whereas others were negative. A BLAST search based on the 250 nt sequence of the PB1 RT-PCR amplicon showed that the influenza gene in the PEBT033 bat was most closely related (77% nt identity) to the PB1 genes of recently described bat influenza viruses, e.g. A/little yellow-shouldered bat/Guatemala/164/2009 (H17N10). The RNA from bat PEBT033 rectal swab sample was analyzed by Sanger and deep sequencing as described previously [2] to generate a full-length genomic sequence (GenBank accession numbers CY125942-CY125949), and this virus was designated A/flat-faced bat/Peru/033/2010 (A/bat/Peru/10) (Table S1).

**Table 1:** Detection of influenza A virus and antibody in bats from Peru (2010).

Species	Location (number captured)	Pan Flu RT-PCR +, tested bats	Seroprevalence +, tested sera
*Artibeus lituratus*	Truenococha (3)	0, 3	3, 3
*Artibeus obscurus*	Santa Marta (1); Truenococha(9)	0, 10	9, 10
*Artibeus planirostris*	Santa Marta (1); Truenococha(14)	1, 15	13, 15
*Carollia brevicauda*	Santa Marta (2)	0, 2	1, 2
*Carollia castanea*	Santa Marta (2)	0, 2	0, 2
*Carollia perspicillata*	Santa Marta (26); Truenococha(4)	0, 29	11, 29
*Desmodus glaucus*	Truenococha(1)	0, 1	0, 1
*Desmodus rotundus*	Santa Marta (12); Truenococha(6)	0, 18	7, 18
*Diphylla ecaudata*	Santa Marta (1)	0, 1	0, 1
*Glossophaga soricina*	Santa Marta (1); Truenococha(3)	0, 2	0, 2
*Molossus molossus*	Santa Marta (10)	0, 10	3, 10
*Myotis sp.*	Santa Marta (6)	0, 6	1, 6
*Phyllostomus discolor*	Santa Marta (1); Truenococha(1)	0, 2	2, 2
*Phyllostomus hastatus*	Truenococha (2)	0, 2	2, 2
*Platyrrhinus recifinus*	Truenococha (1)	0, 1	1, 1
*Rhinophylla pumilio*	Santa Marta (2)	0, 2	1, 2
*Sturnira sp.*	Santa Marta (2)	0, 2	0, 2
*Vampyressa bidens*	Truenococha (3)	0, 2	1, 2

### Evolution of bat influenza viruses

**Figure 1:**
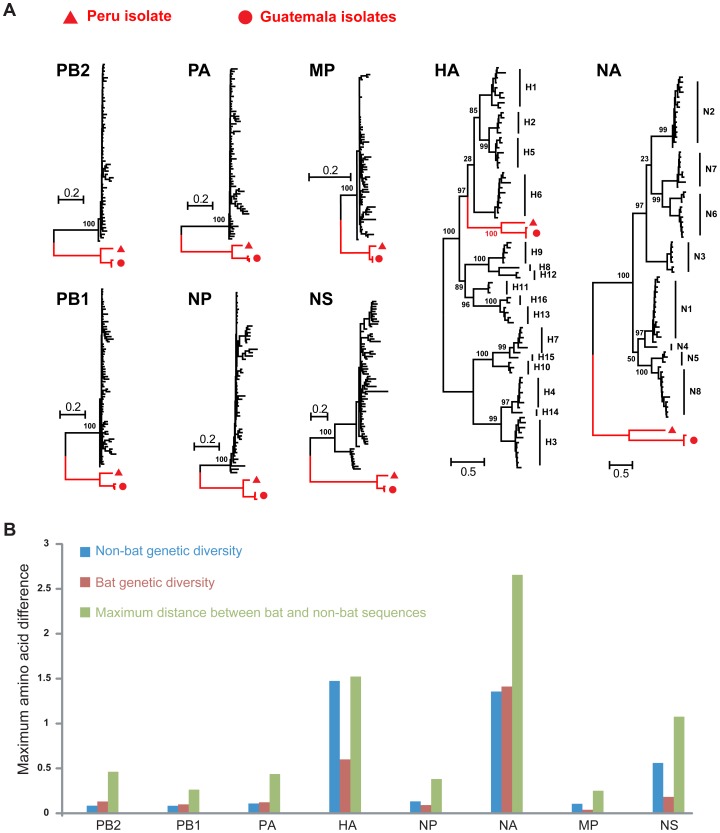
Evolution of influenza virus in New World bats. (a) Phylogenetic relationships of influenza A viruses sampled from bats (red branches) and other animals (‘non-bat’, black branches) based on the amino acid sequences of each gene segment. (b) The maximum amino acid distances are shown within and between the bat and non-bat sequences, and colored accordingly. These levels of diversity also effectively act as a scale-bar for the trees shown above.

Phylogenetic analysis indicated that all genes of A/bat/Peru/10 were most closely related to those of bat influenza viruses from Guatemala, although forming a distinct lineage (Fig. 1). With the notable exception of the HA, the bat virus genes (i) fell as an outgroup to all other known influenza A viruses, and (ii) in four gene segments (PB2, PB1, PA and NA) harbored more genetic diversity than those present in all non-bat (i.e. avian, mammalian) groups combined [2]. Considering the limited geographic area and bat species numbers sampled in the Americas, the remarkable divergence between A/bat/Peru/10 and Guatemalan bat viruses in these four gene segments suggests that New World bat species may carry a diverse pool of influenza viruses. In contrast, the bat HA gene sequences represented a distinct lineage within the known diversity of this gene [2].

The differing phylogenetic position of the bat HA genes relative to the other seven gene segments indicates that a reassortment event took place after gene divergence into the bat and non-bat lineages (Fig. 1). In contrast, the phylogeny of the NA gene resembles that of “internal genes” (PB2, PB1, PA, NP, MP, NS) rather than the HA, an intriguing finding given the functional interdependence of these genes in non-bat influenza viruses (Fig. 1).

Overall, the magnitude of the evolutionary distance between A/bat/Peru/10 and A/bat/Guatemala/164/09 in the HA and NA genes, previously classified as subtype H17N10, would support its designation as an H18N11 virus, comprising new HA and NA subtypes [2]. In particular, these bat viruses are more diverse than some recognized HA and NA subtypes (for example, H13 and H16; N5 and N8; Fig. 1), which is clearly compatible with their classification as distinct subtypes (also see below).

### Genome and proteome of bat influenza viruses

Key structural features of the Peruvian bat influenza virus genome, including RNA transcription and replication promoter elements, open reading frames and mRNA splice signals, and ribosomal frameshift element (PA gene), are nearly identical to the recently described Guatemalan bat influenza genomes and likely to serve equivalent functions [6], [7]. Comparison of the genomes of the two viruses shows nucleotide sequence identity of 48.7–62.3% for genes encoding viral surface proteins and 76.2 to 81.6% for those encoding internal proteins (Table S1).

The influenza HA and NA proteins are critical for interaction with the host and determining the subtype of influenza type A viruses. The mean pairwise amino acid sequence identity of the HA of A/bat/Peru/10 with Group 1 HA subtypes is 49.1% (Table S2). Despite such divergence, canonical sequence motifs conserved across non-bat subtypes of HA, such as putative disulfides, the receptor binding site (RBS), HA0 cleavage sites, coiled-coil heptad repeats and the fusion peptide, are readily identified (Table S3). The HA proteins from A/bat/Peru/10 and the recently identified H17 from Guatemalan bat share only 60.2% sequence identity (Table S2). This divergence is greater than the pairwise differences (Table S2) between 14 of the 136 possible pairwise comparisons between all subtypes, again supporting the designation of the new HA as representative of novel H18 subtype [8]. The NA-like (NAL) protein of A/bat/Peru/10 has only 29.6% identity with all other NA subtypes that correlates with poor residue conservation comprising the canonical catalytic site [9] (Tables S4 and S5) and supports the proposed designation of A/bat/Peru/10 NAL as subtype N11.

The amino-acid sequences of the internal genes of A/bat/Peru/10 retain most of the known functional sequence motifs of other influenza A viruses with few amino-acid substitutions compared to the consensus amino acids at these sites in non-bat viruses (Table 2). To evaluate transcription and replication functions of the A/bat/Peru/10 polymerase complex proteins 4P (PB2, PB1, PA, and NP), we used a minigenome reporter replicon system coupled to transient expression by transfected DNA [10]. In this assay, a mini-genome reporter plasmid, in which luciferase expression is driven by non-coding regions (NCR) from bat (Guat/164.NS-NCR) or human (WSN.NS-NCR) influenza virus, showed high levels of viral transcription when co-transfected with a complete polymerase complex 4P from bat viruses, as compared to control with a plasmid set lacking the PB1 protein (Guat/164-3P) (Fig. S2). The activity of A/bat/Peru/10 RNP complex (Peru/033-4P) with the bat influenza minigenome was 5-fold lower than that of A/bat/Guat/09 (Guat/164-4P), but similar to that of the human (WSN-4P) influenza virus. In contrast, the Peru/033-4P was 16-fold less active than Guat/164-4P with the WSN.NS-NCR (Fig. S2) indicating a possible host restriction for the Peru/033-4P to function efficiently with human influenza genome segments. However, previous reports have shown that differences of this magnitude could be determined by a single amino-acid substitution in one of the polymerase proteins [11], [12].

**Table 2:** Important functional motifs in non-bat influenza A internal proteins are largely conserved in bat influenza proteins.

Protein	Position(s)	Non-bat viruses	A/bat/Guat/09	A/bat/Peru/10	Comment
**PB2**	2–4, 6–7	ERI, EL	_D_RI, EL †		Direct contact with PB1
	627	E or K	S		E associated with avian viruses, K with mammalian viruses
	701	D or N	N		N associated with adaptation to mice
	737–739	RKR			Minor nuclear localization signal
	752–755	KRIR			Major nuclear localization signal
**PB1**	5–10	PTLLFL	P_M_L_I_FL		Binding to PA
	444–446	SDD			Catalytic activity (equivalent to GDD of positive strand viruses)
**PA**	80, 108, 119	E, D, E			Endonuclease catalytic domain
	102, 510	K, H			Cap-snatching activity
	408, 412, 620, 621, 623, 670, 673, 706	Q, N, P, I, E, Q, R, W			PB1 binding
**NP**	3–13	TxGTKRSYxxM	TxG_L_KR_TF_xxM	_G_xGTKR_TF_xxM	Nuclear localization signal
**M1**	101–105	RKLKR	_K_KLK_K_		Nuclear localization signal
**M2**	31	S or N	N		N31 is associated with adamantane resistance
**NS1**	35–41	DRLRR			Nuclear localization signal
	103	F	V		CPSF30 binding/stabilization
	106	M	Q		
	138–147	FDRLETLILL	F_GK_LE_RLV_L_A_	F_GKV_E_RLV_L_A_	Nuclear export signal
	227–230	ESEV or absent	absent		PDZ ligand binding domain
**NS2**	12–21	ILxRMSKMQL			Nuclear export signal

† Letters written as subscripts denote amino acid substitutions in bat viruses compared to the consensus non-bat amino acid sequence.

### HA and NAL protein structure

**Figure 2:**
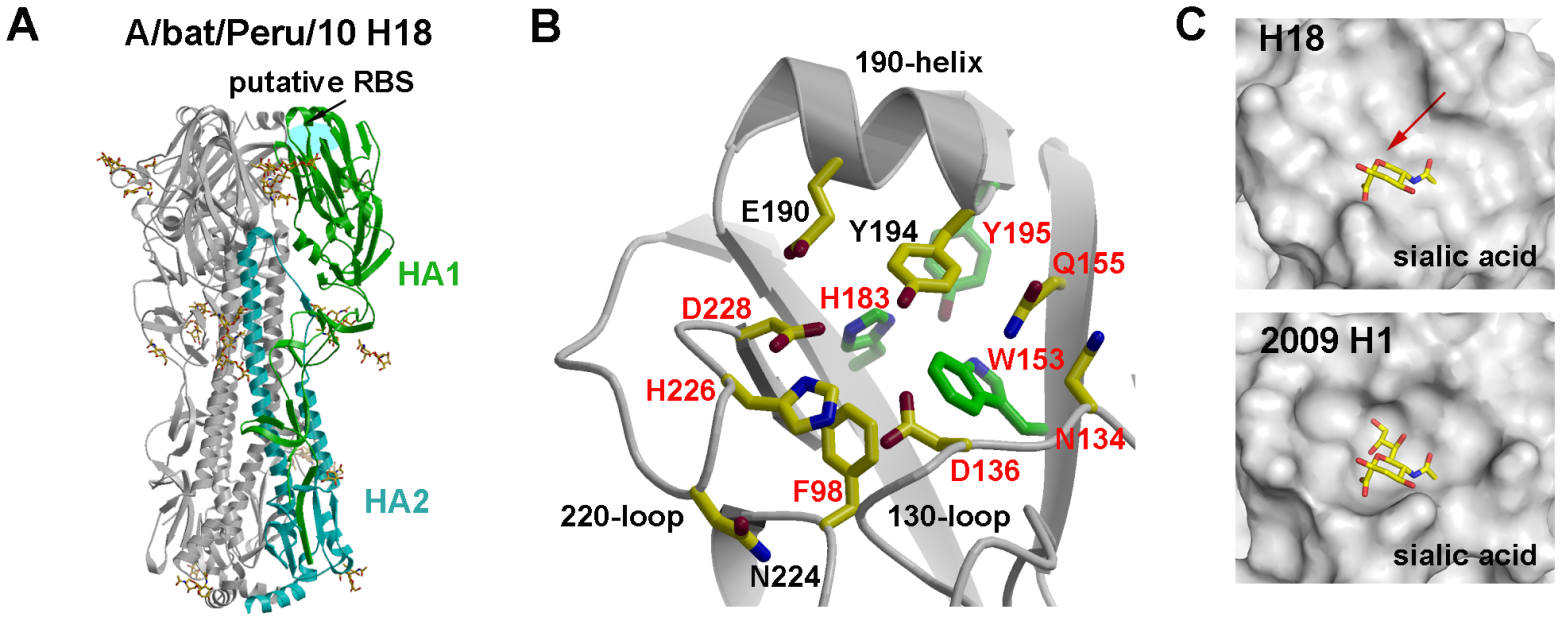
Crystal structures of A/bat/Peru/10 HA. (A) Overall structure of A/bat/Peru/10 HA. The H18 HA trimer consists of three identical monomers with one RBS per monomer. HA1 is highlighted in green and HA2 in cyan. N-linked glycans observed in the electron density maps are shown with yellow carbons. (B) The A/bat/Peru/10 HA putative RBS (in crystal 1, Table S6) in ribbon representation with the side chains of key binding residues shown. The three highly conserved residues (W153, H183, and Y195) in HAs are colored with green carbon atoms, whereas nine residues that are conserved in A/bat/Peru/10 H18 and two H17 HAs from bat influenza viruses A/little yellow-shouldered bat/Guatemala/164/2009 (H17N10) (GU09-164) and A/little yellow-shouldered bat/Guatemala/060/2010 (H17N10) (GU10-060) are labeled in red. E190 and G225 are also conserved, especially in avian influenza A viruses. (C) Molecular surface of the putative RBS of A/bat/Peru/10 HA compared to the RBS of 2009 H1 HA from A/California/04/2009 (H1N1) (PDB code 3UBQ). A canonical sialic acid is modeled in the HA for comparison as observed in other HA structures. The RBS of A/bat/Peru/10 HA is shallower and wider than 2009 H1 HA with no space for the glycerol moiety of sialic acid (indicated by the red arrow). For comparison, figures (B) and (C) are generated in the same orientation.

**Figure 3:**
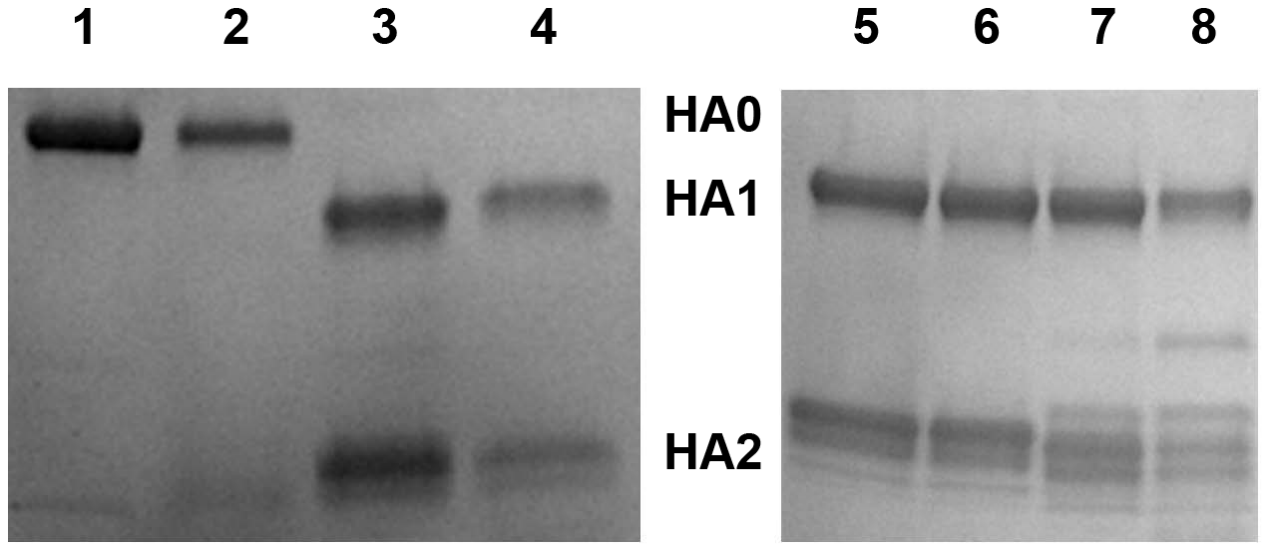
SDS-PAGE of A/bat/Peru/10 HA0 (lanes 1 to 4) and its mature HA (lanes 5 to 8) in trypsin susceptibility assay. A/bat/Peru/10 HA with a monobasic cleavage site was expressed in its HA0 form in a baculovirus expression system. Lanes 1 and 2 show A/bat/Peru/10 native HA0 at pH 8.0 and pH 4.9, respectively, while lanes 3 and 4 show the equivalent reducing gel of HA0 treated with trypsin at pH 8.0 and pH 4.9, respectively. Similarly, lanes 5 and 6 show A/bat/Peru/10 mature HA at pH 8.0 and pH 4.9, respectively, while lanes 7 and 8 show the equivalent reducing gel of mature HA treated with trypsin at pH 8.0 and pH 4.9, respectively.

We determined crystal structures of the A/bat/Peru/10 HA and NAL proteins expressed in a baculovirus system. Two highly similar crystal structures of the A/bat/Peru/10 HA ectodomain were independently determined to 2.15 and 2.24 Å resolution (Table S6) (C_α_ r.m.s.d. of 0.25 Å and 0.34 Å between equivalent monomers and trimers). The overall H18 HA trimer is similar to other HA structures with a membrane-distal globular head comprising the RBS and the vestigial esterase domain, and a membrane-proximal stem region containing the putative monobasic HA1/HA2 cleavage site and fusion peptide (Fig. 2A, Fig. S3, and Tables S3 and S7). The H18 HA ectodomain was expressed in the HA0 form [13]. Tryptic digestion of HA0 at pH 8.0 resulted in specific cleavage to HA1/HA2 (Fig. 3), suggesting A/bat/Peru/10 HA also requires processing to be functional for infection. Surprisingly, however, trypsin digestion at pH 4.9 did not degrade HA1 and HA2 (Fig. 3) in contrast to other HAs that adopt a fusion active form on exposure to pH≤5.5 exposing trypsin cleavage sites and degradation in this assay. Thus, although the fusion peptide, coiled-coil heptad repeats, HA2 Arg106, and the stem region in general are largely conserved, the requirements for the pH-induced conformational change that occurs upon activation of membrane fusion appear to be different [14], [15]. The membrane-distal domain mediates receptor binding and contains most of the epitopes recognized by antibodies. The HAs from influenza viruses are well documented to bind to glycan receptors with terminal sialic acid. The RBS in influenza A contains several highly conserved amino acids at its base: Tyr98, Trp153, His183, and Tyr195 (H3 numbering, Tables S3 and S8), and three major structural elements at its edge: 130-loop (134–138), 220-loop (221–228), and 190-helix (188–194) [16], [17]. In all HAs examined to date, sialic acid binds through hydrophobic interactions and hydrogen bonds with HA residues from the 130- and 220-loops and 190-helix (Fig. S3C) as well as the base [18]. In contrast, H18 HA has a dramatic alteration of the binding site residues (Fig. 2B, Tables S3 and S8) where only three (Trp153, His183, and Tyr195) of the key RBS residues in influenza A viruses [16], [19] are conserved (Fig. 2B, Table S8), although Glu190 and Gly225 represent consensus sequences in avian HAs. Tyr98 that hydrogen bonds to the 8-hydroxyl group of sialic acid in influenza A viruses, is replaced in H18 by Phe98 (Table S8). Significantly, Leu194 in influenza A and B viruses, another critical residue for sialic acid binding [20], is replaced by Tyr194 in H18 (Table S8). Together with other larger residues, including Asp136, Gln155, and Asp228, which are unique to H18 and H17 HA bat proteins (Tables S3 and S8), the addition of Tyr194 dramatically flattens and widens the RBS as compared to pandemic 2009 H1 (Fig. 2C) and other HAs (Fig. S4). The glycerol side chain of a canonical sialic acid would clash with Tyr194 and Asp228 (Fig. 2C). In addition, Asp136, which is not found in H1–H16, would electrostatically repulse the sialic acid carboxylate. Several assays were used to investigate the binding of the H18 HA to mammalian glycans. H18 HA exhibited no specific binding to a custom sialoside microarray (data not shown) [9], [21] or the glycan microarray of the Consortium for Functional Glycomics (CFG) that contains 610 diverse glycans found on mammalian cells, including over 100 unique sialosides (*α*2-3, *α*2-6,, *α*2-8, and mixed linkages) (Fig. S5A and Tables S9, S10) [22]. In contrast, the influenza H5 HA bound robustly to numerous sialosides on the array (Fig. S5C). The lack of binding to sialosides was further confirmed in a plate-based ELISA with glycans 3′-SLNLN and 6′-SLNLN (Fig. S5D–E) showing no detectable binding to either sialoside, in contrast to H5 HA. These data strongly suggest that bat H18 HA does not recognize sialic acid receptors, and its receptor remains to be defined.

**Figure 4:**
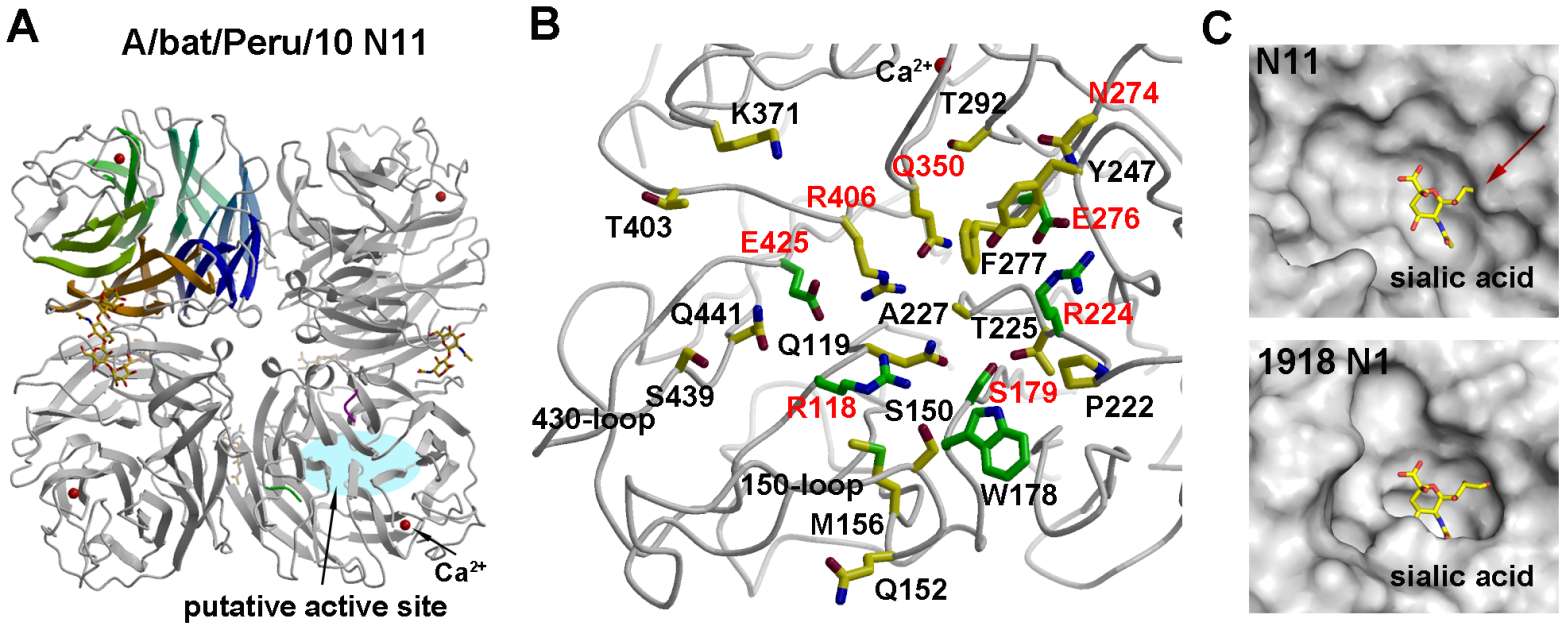
Crystal structures of A/bat/Peru/10 NAL. (A) Overall structure of A/bat/Peru/10 NAL with a conserved calcium binding site. The N11 NAL tetramer is viewed from above the viral surface, and consists of four identical monomers with C4 symmetry. One monomer is colored in six different colors to illustrate the canonical *β*-propeller shape of six four-stranded, anti-parallel *β*-sheets. The putative active site is located on the membrane-distal surface (on top of the molecule). The observed N-linked glycosylation sites are shown with attached carbohydrates. A single calcium ion is shown in red spheres. (B) The A/bat/Peru/10 NAL putative active site (crystal form 1, Table S6) with the conserved catalytic and active site residues in other NAs shown as well as other polar and charged residues. The six residues (R118, W178, S179, R224, E276 and E425) conserved in all influenza NAs are colored with green carbon atoms in contrast to other putative active site residues in yellow carbon atoms, whereas eight residues that are conserved in two bat influenza N10 and N11 NAL proteins are labeled in red. (C) Molecular surface of the active site of A/bat/Peru/10 N11 NAL and 1918 N1 NA from A/Brevig Mission/1/18 (H1N1) (PDB code 3BEQ). A canonical sialic acid is modeled in A/bat/Peru/10 NAL as in other NA structures and appears to collide with the NA putative active site around the glycerol moiety (as indicated by the red arrow). The putative active site pocket of A/bat/Peru/10 NAL is much wider than 1918 N1 NA. For comparison, figures (B) and (C) are generated in the same orientation.

The extensive sequence divergence of A/bat/Peru/10 NAL from the recently characterized N10 prompted us to determine its X-ray structure in two crystal forms at 2.68 Å and 3.0 Å (Fig. S6A, Table S6). The N11 NAL forms a typical “box-shaped” tetramer, containing six four-stranded, antiparallel *β*-sheets in a propeller-like arrangement (Fig. 4A). Comparison of the A/bat/Peru/10 NAL monomer with other NA structures reveals surprising similarity despite low sequence identity (29.6%) (Fig. S6B–D and Table S4). In addition, a calcium required for stabilization of all known influenza A and B NA active sites [23], [24] is conserved in N11 NAL (Fig. 4A). Taken together, the N11 NAL and the N10 NAL [9] are homologous proteins characterized by highly diverged putative active sites (Fig. 4B). Among the eight conserved charged and polar residues that interact with substrate in all other influenza A and B subtypes [25], only Arg118, Arg224, Glu276 are conserved (N2 numbering, Fig. 4B, Tables S5 and S11) as in N10 NALs [9]. Arg292 and Arg371, critical for sialic acid binding, are replaced in A/bat/Peru/10 NAL by Thr and Lys, respectively. Furthermore, only three of eleven second-shell residues are conserved in N11 NAL (Trp178, Ser179 and Glu425) (Fig. 4B, Tables S5 and S11). The N11 NAL active site pocket is much wider than other flu A and B NAs, including 1918 N1, due mainly to 150- and 430-loop movements (Fig. 4C) [26]. The glycerol moiety of a canonical sialic acid, as observed in other flu A and B NA structures [26], would clash with the N11 NAL active site (Fig. 4C). In comparison with bat N10 NAL [9], eight residues are conserved in the putative active site (Fig. 4B, Table S11). N11 NAL catalyzes extremely low levels of sialic acid cleavage (Fig. S7) [22] as observed for N10 NAL [9], and similar to H18 HA, N11 NAL exhibited no binding to 610 diverse natural glycans including sialosides in a glycan microarray assay (Fig. S5B and Tables S9, S10). Thus, neither H18 HA nor N11 NAL appears to bind sialic acids, suggesting they interact with receptors that have still to be identified to retain their function of mediating entry and release of bat H18N11 viruses.

### Influenza virus infections in bats

To assess the seroprevalence of infection in South American bat populations, we analyzed panels of sera to identify IgG antibodies to recombinant bat influenza H18 HA (rHA) and N11 NAL (rNAL) by indirect ELISA. Specific IgG antibody titers to bat rHA or rNAL (>1∶1,000) were detected among 55 of 110 bats from Peru (Table 1). High titers (≥1∶16,000) to rHA or rNAL were detected in 11 sera (10%). A high proportion of these 55 bat sera (21 samples) were positive for both rHA and rNAL, whereas 30 were positive for rHA only and 4 positive for rNAL only. A selection of H18 positive sera were also tested against H17, H1 and H5 (rHAs), and no cross reactivity was detected, in agreement with the proposed H18 subtype designation (Fig. S8). If the immunodominance of HA in humans and swine is recapitulated in bats, the absence of antibodies to HA in NAL-seropositive animals suggests that bat viruses of an unknown HA subtype, in combination with N11, may have infected these animals [27]. The proportion of seropositive samples was highest among *Artibeus* in Truenococha (25 of 28 tested, positive for rHA or rNA). Five additional bat species also appear to be highly seropositive despite small sample sizes (Table 1).

The high seroprevalence of bat influenza in bats from the Loreto Department in Peru prompted analysis of 228 serum samples from eight locations in southern Guatemala in 2009–2010. Specific antibodies to bat H17 subtype rHA were detected by ELISA in 86 of the 228 (38%) sera from eight bat species (Table S12). The temporal and spatial limitations of our sampling notwithstanding, the high seroprevalence of influenza virus infection in multiple species suggests widespread circulation of influenza A viruses among New World bats.

## Discussion

We have characterized a new influenza virus from a flat-faced fruit bat (*Artibeus planirostris*) in the Amazon River basin in northern Peru. While our phylogenetic analysis indicated that the Peruvian bat virus shares common ancestry with recently identified Guatemalan H17N10 bat viruses, their genetic and phylogenetic divergence is such that we propose that their HA and NA genes be designated as new subtypes H18 and N11, respectively. A high frequency of antibodies is consistent with widespread circulation of these viruses in bat populations from the Americas.

The crystal structure and glycan array analysis of A/bat/Peru/10 HA indicate that sialic acid is not the receptor for attachment to host cells, consistent with similar analyses of bat NAL that showed substitution of catalytic residues and no sialidase activity in recombinant bat NAL preparations [9]. Hence, these data suggest that bat influenza HA and/or NAL mediate host cell entry and release via different receptors compared to other influenza viruses. More generally, these discoveries highlight the functional plasticity of influenza A viruses. In addition, attempts to propagate this virus in mammalian and avian cell cultures have been unsuccessful, although viral transcription from reporter minigenomes is functional in human and primate cells. This complicates the development of animal models to investigate the host range and virulence of bat influenza viruses. Taken together, these obstacles create significant gaps in our knowledge for assessing the potential public health significance of these viruses. Until these problems are resolved, serologic studies in human populations that may come in contact with bats in Central or South America that potentially harbor H17N10 and H18N11 influenza viruses may help inform risk assessment efforts.

Multiple lines of evidence suggest that influenza viruses have evolved in bats for an extended period of time: (i) in four of the eight gene segments, genetic diversity exceeds that observed in all other animal species combined; (ii) the divergence into multiple HA subtypes and utilization of alternative mechanisms for sialic acid-independent virion attachment to target cells and subsequent release; and (iii) the widespread geographic distribution in the Americas (Guatemala and Peru sampling sites are ∼3,500 km apart) combined with a high seroprevalence in several bat species are indicative of an established infection, while the observation that the bat viruses form a monophyletic group is suggestive of sustained transmission in this species. The postulated ancient relationship of influenza viruses with aquatic migratory birds is consistent with the optimized parasitic relationship of the virus with ducks, involving subclinical infections with multiple virus subtypes as a result of efficient fecal-oral transmission. Although necessarily preliminary in nature, the data presented here suggest that similar ecological and evolutionary strategies may have been exploited by the influenza A viruses of New World bats.

## Materials and Methods

### Ethics statement

All procedures reported herein were performed in accordance with institutionally approved animal care and use protocols (1843 RUPMULX approved by the Centers for Disease Control and Prevention Institutional Animal Care and Use Committee). All aspects of the bat collections were undertaken with the approval of the a Peru Ministry of Agriculture (permit RD-0389-2010-DGFFS-DGEFFS) and following the American Veterinary Medical Association guidelines on euthanasia and the Guidelines of the American Society of Mammalogists for the use of wild mammals in research.

### Sample collection

CDC field sampling of selected mammals, such as bats, has been ongoing for several years as a means of background zoonotic surveillance for primary pathogen detection [28]. Field work was related to a reported high incidence of vampire bat depredation to human communities in the Peruvian Amazon [29]. Bat sampling was conducted for enhanced rabies surveillance related to concurrent human surveys, and to improve an understanding of pathogen diversity in the Neotropical bat fauna. Bats were captured manually and by using mist nets and hand nets; adults and subadults of both sexes were captured. Bats were restrained, sedated, euthanized, and a complete necropsy was performed in compliance with established field protocols. A total of 114 bats from at least 18 different species were captured from Truenococha and Santa Marta, two communities in the Loreto Department of Peru at the edge of the Amazon River basin (Fig. S1). Representative tissues were removed from bats. Samples of serum, tissues, organs, rectal and oral swabs were immediately stored in liquid nitrogen in the field and then at −80°C in the laboratory until shipment to CDC for processing and analysis. Total nucleic acids (TNA) were extracted from 200 µL of a phosphate buffered saline suspension of each swab by using the QIAamp MinElute Virus Spin kit (QIAGEN, Santa Clarita, CA), according to the manufacturer's instructions and then stored at −80°C.

### Pan-influenza reverse transcriptase PCR

TNA extracted from the rectal swabs (n = 110) were screened for the presence of influenza virus RNA using pan-influenza (pan-Flu) reverse transcriptase PCR (RT-PCR) as described previously [2]. Positive and negative RT-PCR controls containing standardized viral RNA extracts and nuclease-free water were included in each run. Standard precautions were taken to avoid cross-contamination of samples before and after RNA extraction and amplification. Each of the positive results was repeated and confirmed from different TNA aliquots of the original bat rectal swab eluate. The resulting PCR amplicons were separated by electrophoresis in agarose gel and purified using QIAquick PCR Purification kit or QIAquick Gel Extraction kit (Qiagen, Santa Clarita, CA). Purified DNA amplicons (both strands) were then sequenced with the pan-Flu RT-PCR primers on an ABI Prism 3130 automated capillary sequencer (Applied Biosystems).

### Complete genome sequencing

The pan-Flu RT-PCR positive rectal swab suspension was subjected to both high throughput next generation sequencing and RT-PCR amplicon-based Sanger sequencing as described previously [2]. In brief, 200 µl of rectal swab suspension (in PBS) from the bat PEBT033 was first cleared through a 0.22-µm Ultrafree-MC filter (Millipore) and then extracted using the QIAamp MinElute Virus Spin kit. The extracted TNA was randomly amplified using the Round AB protocol as previously described [2]. Amplification products were subjected to high-throughput sequencing by an Illumina GAIIx (Illumina) at Emory University. The resulting sequence was extracted and de-multiplexed using Illumina SCS2.8 software. The data were then analyzed using the CLC Genomics Workbench package. The imported reads were trimmed to remove low quality sequence as well as any reads of ≤36 bases in length. The reads were assembled *de novo* with a minimum contig length of 75 bases. All contigs with a coverage depth ≥3X where submitted to BLASTn against the non-redundant (nr) NCBI database to identify influenza sequences. This process was repeated with tBLASTx to find segments that were not identified from nucleotide BLASTn. To increase the reliability of the sequence data from Illumina sequencing, the rectal swab of bat PEBT033 was also processed by Sanger sequencing on RT-PCR amplicons of genome segment. The viral genome was amplified directly from the TNA extracted from bat PEBT033 rectal swab suspensions using universal influenza A primers (FWuni12 and RVuni13) [30]. The 800 bp to 2.3 kb amplicons were then cloned using the pCR-XL-TOPO TA cloning kit (Invitrogen). Eight to 16 colonies from each of the 8 segments' RT-PCR transformation were first sequenced with M13 forward and reverse primers in both directions, and the remaining internal gaps were sequenced with sequence-specific walking primers in both directions. The 3′ end and 5′ end sequences of each segment from bat PEBT033 were determined using the 5′/3′ RACE kit (Roche) according to the manufacturer's instructions. Sequence analysis and generation of contigs were performed using Sequencher software. Consensus gene sequences were compared to those from the high throughput next generation sequencing methods. Sequence identification was performed through NCBI BLASTn and tBLASTx similarity searches.

### Sequence data set

8486 complete genome sequences of influenza A virus, comprising both avian and other mammalian hosts, were downloaded from the GISAID database (http://platform.gisaid.org/epi3/frontend). Sequence alignment was performed on the amino-acid sequences of each gene segment using MAFFT v6.853b [31], [32]. Because of the highly divergent nature of the HA and NAL segments, all ambiguously aligned sites in these segments were removed using the Gblocks program [33]. This resulted in final alignment lengths for the HA and NAL proteins of 507 and 395 amino acids, respectively. Because of the very large number of sequences available from some subtypes, each amino acid alignment was further subsampled based on sequence similarity to obtain smaller data sets containing between 300 and 400 representative sequences. Pairwise genetic distances were then estimated between these sequences using the JTT model of amino acid substitution available in the MEGA5 v5.05 package [34].

### Phylogenetic analyses

To assist our phylogenetic analyses of these sequence data, we further reduced the sample size to 50–70 representative sequences for each gene segment. Phylogenetic trees of these data were then estimated using the maximum likelihood (ML) method available in the PhyML package [35], employing 100 bootstrap replicates. In all cases, the JTT model of amino acid substitution was employed with four categories of gamma-distributed rate heterogeneity and a proportion of invariant sites (JTT+Γ_4_+I).

### ELISA assay

An indirect ELISA using bat influenza neuraminidase and hemagglutinin was run to establish the seroprevalence of IgG antibodies to bat influenza within the Peru bat population. In brief, ELISA plates were coated with recombinant bat hemagglutinin (rHA18, rHA17), recombinant avian hemagglutinin (rHA5 and rHA1), or recombinant bat neuraminidase-like (rNAL) at a concentration of 1 µg/mL (100 µL per well) in PBS, pH 7.4 overnight at 4°C. The next morning, bat sera were heat inactivated at 56°C for 30 minutes. Plates were washed 3 times with PBS with Tween 20 (0.1%), pH 7.4 (PBST). Bat sera were diluted 1∶500 in 2.5% non-fat milk-PBST and added to each well in duplicate, two-fold dilutions. Plates were incubated for 1 hour at 37°C. The test sera were removed and the plates washed 3 times with PBST. Biotinylated protein G (MBL corp) (a 1∶1,000 dilution of 1 mg/mL solution, 100 µL) was added to each well and the plates were incubated at 37°C for 1 hour. The plates were washed 3 times with PBST. Streptavidin HRP (Millipore) (a 1∶10,000 dilution of 1 mg/mL solution, 100 µL) was added to each well and the plates were incubated at 37°C for 1 hour. The plates were washed 3 times with PBST and O-phenylenediamine (OPD), with H_2_O_2_ and phosphate-citrate buffer, was added to each well (100 µL). The plates were incubated at 37°C for 10 minutes, the color change was stopped by addition of 100 uL of 3N HCl, and the plates read by a plate reader. The limit of detectable response (based on 490 nm at 0.1 s OD) for the ELISA was set as values above average background plus 2 standard deviations.

### Cloning, expression and purification of A/bat/Peru/10 H18 HA protein used for crystal 1

The ectodomain (residues 15-513, equivalent to 11-329 of HA1 and 1-174 of HA2 in H3 numbering) of the H18 protein from influenza virus A/flat-faced bat/Peru/033/2010 (H18N11, GenBank accession number CY125945) was expressed in a baculovirus system for structural and functional analyses. The cDNA corresponding to the H18 HA ectodomain was inserted into a baculovirus transfer vector, pFastbacHT-A (Invitrogen) with an N-terminal gp67 signal peptide, a C-terminal thrombin cleavage site, a foldon trimerization sequence, and a His_6_-tag [21]. The constructed plasmids were used to transform DH10bac competent bacterial cells by site-specific transposition (Tn-7 mediated) to form a recombinant Bacmid with beta-galactosidase blue-white receptor selection. The purified recombinant bacmids were used to transfect Sf9 insect cells for overexpression. The HA protein was produced by infecting suspension cultures of Hi5 cells with recombinant baculovirus at an MOI of 5–10 and incubated at 28°C shaking at 110 RPM. After 72 hours, Hi5 cells were removed by centrifugation and supernatants containing secreted, soluble HAs were concentrated and buffer-exchanged into 20 mM Tris pH 8.0, 300 mM NaCl, further purified by metal affinity chromatography using Ni-nitrilotriacetic acid (NTA) resin (Qiagen), For crystal structure determination, the HA ectodomain was digested with thrombin to remove the foldon domain and His_6_-tag. The cleaved trimeric H18 HA ectodomain was purified further by size exclusion chromatography on a Hiload 16/90 Superdex 200 column (GE healthcare) in 20 mM Tris pH 8.0, 100 mM NaCl and 0.02% NaN_3_.

### Cloning, expression and purification of A/bat/Peru/10 N11 NAL protein used for crystal form 1

The cDNA corresponding to the ectodomain of A/bat/Peru/10 NAL (residues 83-448, equivalent to 81-459 in N2 numbering) from influenza virus A/flat-faced bat/Peru/033/2010 (H18N11, GenBank accession number CY125947) was cloned into the baculovirus transfer vector pAcGP67-B, in frame with an N-terminal cassette containing a His_6_-tag, a vasodilator-stimulated phosphoprotein (VASP) domain and thrombin cleavage site [36]. Transfection and virus amplification were carried out as described previously [10]. Protein expressed from *Sf9* cells (Invitrogen) in 3-liter shaking flasks (Corning Inc.) was recovered from the culture supernatant and purified by metal affinity chromatography, subjected to thrombin cleavage and gel filtration chromatography. The purified monomeric N11 NAL protein was buffer exchanged into 10 mM Tris-HCl, 50 mM NaCl, pH 8.0 with 1 mM CaCl_2_ and concentrated to 14 mg/ml for crystallization trials.

### Crystal structure determination of A/bat/Peru/10 H18 HA in crystal 1

Crystallization experiments were set up using the sitting drop vapor diffusion method. The A/bat/Peru/10 HA ectodomain trimer protein at 14 mg/ml in 20 mM Tris pH 8.0, 100 mM NaCl and 0.02% (w/v) NaN_3_ was crystallized in 0.1 M MES, pH 6.9, 5% (w/v) polyethylene glycol (PEG) 1000 and 27.5% (w/v) PEG 600 at 22°C. The A/bat/Peru/10 HA crystals were flashed-cooled at 100 K without additional cryo-protectant. Diffraction data were collected at beamline 23ID-D at the Advanced Photon Source (APS) (Table S6). Data for all crystals were integrated and scaled with HKL2000 [37].

The A/bat/Peru/10 HA structure was determined by molecular replacement (MR) using the program Phaser [38] with A/bat/Peru/10 HA from its crystal structure in complex with an antibody Fab as the search model. This Fab complex structure was previously determined using A/South Carolina/1/18 (H1N1) H1 HA (PDB code 1RD8) as the MR search model and will be published elsewhere. Initial rigid body refinement of A/bat/Peru/10 HA was performed in Refmac5 [39], and simulated annealing and restrained refinement (including TLS refinement) were carried out in Phenix [40]. Between rounds of refinements, model building was carried out with the program Coot [41]. Final data processing and refinement statistics are represented in Table S6. The quality of the structure was determined using the JCSG validation suite (www.jcsg.org). All figures were generated with Bobscript [42] except for Figs. 2C and 2F, Figs. S3 and S4, which used PyMol (www.pymol.org).

### Protease susceptibility assay

Each trypsin cleavage reaction containing ∼2.5 µg of A/bat/Peru/10 HA0 or trypsin-digested A/bat/Peru/10 mature HA (HA1/HA2) and 1% dodecylmaltoside (to prevent possible aggregation with any post-fusion HA) was set up at room temperature (∼22°C). Sodium acetate was used as the pH 4.9 buffer and Tris was used as the pH 8.0 buffer. Reactions were thoroughly mixed, centrifuged at >12,000 g for 30 seconds and allowed to incubate at 37°C for one hour. After incubation, reactions were equilibrated to room temperature and the pH was neutralized by addition of 200 mM Tris, pH 8.5. Trypsin was added to all samples, except controls, at a final ratio of 1∶1 (w/w) for A/bat/Peru/10 HA. Samples were digested overnight (∼18 hours) at 22°C. Reactions were quenched by addition of non-reducing or reducing SDS buffer and were boiled for ∼2 min. Samples were analyzed by SDS-PAGE.

### Crystal structure determination of A/bat/Peru/10 N11 NAL in crystal form 1

Initial crystallization trials were set up using a Topaz™ Free Interface Diffusion (FID) Crystallizer system (Fluidigm Corporation). Crystals were observed in conditions containing various molecular weights of PEG polymer. Following optimization, diffraction quality crystals for A/bat/Peru/10 N11 NAL were obtained at 20°C using a sitting drop method. The crystallization condition was 0.2 mM calcium acetate, 10% (w/v) PEG 8000, 0.1 M HEPES at pH 7.5. Crystals were flash-cooled at 100 K using 20% (v/v) glycerol as a cryoprotectant. Data were collected at the Advanced Photon Source (APS) beamline 22-ID at 100 K and processed with the DENZO-SCALEPACK suite [37].

The structure of A/bat/Peru/10 NAL was determined by molecular replacement with Phaser [38] using the N10 NAL structure from A/little yellow-shouldered bat/Guatemala/164/2009 (H17N10) (PDB code 4GEZ) as the search model (sequence identity is 40%). Four neuraminidase monomers were found that constituted one non-crystallographic tetramer with an estimated solvent content of 63.6% based on a Matthews' coefficient (*Vm*) of 3.38 Å^3^/Da. The model was rebuilt by Coot [41] and structure refined with Refmac5 [43]. The final model was assessed using MolProbity [44]. Statistics on data processing and refinement are presented in Table S6.

### Cloning, expression, purification, and crystal structure determination of A/bat/Peru/10 surface proteins in crystal form 2

Methods used for H18 HA and N11 NAL proteins used for crystal form 2 were as described (see Text S1).

### Neuraminidase activity assay with substrate 4-MU-NANA

NA enzymatic activities were measured by using fluorescent substrate 2′-(4-methylumbelliferyl)-α-D-*N*-acetylneuraminic acid (4-MU-NANA) [45] in 100 mM imidazole-malate pH 6.15, 150 mM NaCl, 10 mM CaCl_2_ 0.02% NaN_3_ buffer with excitation and emission wavelengths of 365 nm and 450 nm, respectively. The reaction was conducted for 60 minutes at 37°C in a total volume of 80 µl for the N11 NAL protein with ectodomain plus stalk region that was expressed as a tetramer. The reactions were all performed in triplicate and were stopped by adding 80 µl of 1 M Na_2_CO_3_. To compare NA cleavage activities at 12 different NA concentrations, with a fixed substrate 4-MU-NANA concentration of 0.05 mM, the NA starting solution at 0.51 mg/ml was serially diluted 1∶2.

### Data sharing

The sequence data reported in this paper were deposited in the GenBank database at NCBI under accession numbers CY125942-CY125949. The atomic coordinates and structure factors of the A/bat/Peru/10 H18 crystals 1 and 2, and A/bat/Peru/10 N11 in crystal forms 1 and 2 are being deposited in the Protein Data Bank, www.rcsb.org (PDB ID codes 4K3X, 4MC5, 4MC7 and 4K3Y).

## Supporting Information

Figure S1 Geographic locations of bat-sampling sites in Peru. Bats were captured at Truenococha and Santa Marta in the Loreto District (the inset depicts this region within the Republic of Peru, see Table 1 and Table S1 for additional information).
(PDF)

Figure S2 RNA-dependent RNA polymerase activity of A/bat/Peru/10 ribonucleoprotein (RNP) complex proteins (PB2, PB1, PA and NP). A549 human lung cells were transfected with pPol1-A/bat-NS.NCR-Renilla (Guat/164.NS-NCR) or pPol1-A/WSN-NS.NCR-Renilla (WSN.NS-NCR) and pSV40-Luc reporter plasmids, together with plasmids expressing PB2, PB1, PA and NP from either A/WSN/33 (WSN-4P) or A/bat/Peru/10 (Peru/033-4P) or A/bat/Guat/09 (Guat/164-4P) viruses or without the PB1 expression plasmid (Guat/164-3P). Values shown represent the activities of each RNP and reporter relative to that of WSN virus (100%) with the homologous reporter. Error bars indicate 95% confidence intervals. Experiments were performed three times independently.
(PDF)

Figure S3 The structure of A/bat/Peru/10 HA in crystal 2. (A) One monomer is shown with the HA1 chain colored in green and the HA2 chain in cyan. The glycosylation positions are highlighted in magenta with the glycan in yellow. A/bat/Peru/10 HA has four potential N-linked glycosylation sites in HA1 (Asn21, Asn242, Asn264, Asn289) and a further two in HA2 (Asn145 and Asn154). While position 21 is close to the HA1/HA2 cleavage site, position 242 is closer to the putative receptor binding pocket. Positions 264 and 289 are close together in the middle of the molecule around the vestigial esterase domain in HAs from other influenza A viruses. In the HA2, positions 145 and 154 are near the membrane-anchoring region. Asn154 is conserved in all HAs, while Asn145 is only found in HA sequences from three other group 1 subtypes (H13, H16 and H17). From these structures, interpretable electron density for one or two N-acetyl glucosamines was observed at all six of these putative glycosylation sites. However, due to crystal packing, density for the carbohydrate at Asn242 was well defined in all three monomers and could be visualized up to the first three mannoses of the glycan. (B) The putative receptor binding site with the three structural elements, the 130-loop, 220-loop and the 190-helix. The putative binding site residues are shown in sticks. (C) Superposition of receptor binding site region of A/bat/Peru/10 H18 (in green), 1918 H1 (in salmon, PDB code 1RD8), 2009 H1 (in purple, 3M6S), swine H1 (in pink, 4F3Z), human H2 (in grey, 2WR7), human H5 (in yellow, 2FK0), swine H9 (in orange, 1JSD), human H3 (in cyan, 2HMG), human H7 (in marine, 4DJ6) and mallard H14 (in slate, 3EYJ).
(PDF)

Figure S4 Comparative surface representation of the receptor binding sites of bat and non-bat HAs. A/bat/Peru/10 H18 HA (in green), 1918 H1 HA (salmon, PDB code 1RD8), 2009 H1 HA (purple, 3M6S), swine H1 HA (pink, 4F3Z), H2 HA (grey, 2WR7), H5 HA (yellow, 2FK0), H9 (orange, 1JSD), H3 HA (cyan, 2HMG), H7 HA (marine, 4DJ6) and H14 HA (slate, 2EYJ), with arrows indicating the receptor binding sites in other HAs.
(PDF)

Figure S5 Glycan binding analysis of A/bat/Peru/10 HA and NAL. (A to C) Glycan microarray analysis of A/bat/Peru/10 HA (A) and NAL (B), and control protein A/Vietnam/1203/2004 H5 HA (C) was performed on the CFG glycan microarray v5.1, which contains 610 mammalian glycans. Binding signals (black bars) are shown in relative fluorescence units (RFU). The H5 HA showed good binding avidity to *α*2-3 glycans, but A/bat/Peru/10 HA and NAL exhibit no specific binding to any glycans on the array, including natural sialosides with *α*2-3, *α*2-6, *α*2-8 and mixed linkages, and other glycans that might exist in mammals (see Tables. S9 and S10). (D to E) ELISA-based plate assay of A/bat/Peru/10 HA and control A/Vietnam/1203/2004 H5 to 3′-SLNLN (D) and 6′-SLNLN (E). The H18 HA does not bind to either 3′-SLNLN or 6′-SLNLN in the experimental conditions, while the control H5 HA binds more strongly to 3′-SLNLN as expected.
(TIF)

Figure S6 Stereo view of superimposed A/bat/Peru/10 NAL in crystal form 1 (in grey) with other NA structures. (A) Comparison with A/bat/Peru/10 NAL in crystal form 2 (in green) with C_α_ r.m.s.d. of 0.7 Å. The structures of A/bat/Peru/10 NAL in two crystal forms are the same except for the 150-loop, which is flexible in crystal form 2 and not modeled. (B) Comparison with GU09-164 NAL in crystal form 1 (in sky blue, PDB code 3GDI) with r.m.s.d. of 1.3 Å. The overall structures are very similar except for 150-loop which is closer to the putative active site in A/bat/Peru/10 N11 NAL, making the putative active site less open. (C) Comparison with GU09-164 NAL in crystal form 2 (in magenta, PDB code 3GEZ) with C_α_ r.m.s.d. of 1.2 Å. The overall structures are very similar except for 150-loop which is further from the putative active site in A/bat/Peru/10 N11 NAL, making the putative active site more open. (D) Comparison with 1918 N1 NA (in purple, PDB code 3BEQ) with C_α_ r.m.s.d. of 1.7 Å. As large conformational changes are observed in the 110-loop, 150-loop and 430-loop, as well as C-terminus, these regions were excluded from the RMSD calculation. Compared to 1918 N1 NA, the A/bat/Peru/10 NAL putative active site in crystal form 1 adopts a much more open conformation.
(PDF)

Figure S7 NA cleavage activity analysis of A/bat/Peru/10 NAL. Only extremely low sialic acid cleavage activity was observable with NAL concentrations as high as 100 µg/ml.
(PDF)

Figure S8 Detection of antibody to HA subtypes in bat sera by ELISA. Plates were coated with 1 ug/mL of H18 rHA antigen (homologous), or H17, H5,H1 rHA antigen (heterologous) to ascertain levels of cross reactivity. Serial log_2_ dilutions of sera were performed, starting at 1∶500, and an absorbance reading was taken at 490 nm for 0.1 seconds. Samples represented are (A) Peru 017, (B) Peru 019, (C) Peru 020, and (D) Peru 031.
(PDF)

Table S1 Nucleotide sequence identity between A/flat-faced bat/Peru/033/2010 (H18N11) and A/little yellow-shouldered bat/Guatemala/164/2009 (H17N10) genomes.
(DOCX)

Table S2 Mean amino acid identity between A/bat/Peru/10 H18 HA and influenza A subtypes H1–H17.
(DOCX)

Table S3 Sequence comparison after sequence and structural alignment of HA1 (top) and HA2 (bottom) of A/bat/Peru/10 H18 HA with bat H17 HAs and non-bat HAs ^a^.
(DOCX)

Table S4 Mean amino acid identity between the A/bat/Peru/10 N11 NAL and representative NAs of influenza A and B viruses.
(DOCX)

Table S5 Sequence comparison of A/bat/Peru/10 N11 NAL with bat N10 NALs and other influenza NAs^a^.
(DOCX)

Table S6 Data collection and refinement statistics of A/bat/Peru/10 HA and A/bat/Peru/10 NAL.
(DOCX)

Table S7 Comparison of C_α_ rmsd values (Å) of A/bat/Peru/10 HA (crystal 1) with other influenza A virus HAs^a^.
(DOCX)

Table S8 Conservation of key residues in influenza A HA receptor-binding site ^a^.
(DOCX)

Table S9 List of glycans on the microarray.
(XLS)

Table S10 List of 25 glycans that give strongest binding signals in the microarray.
(XLSX)

Table S11 Conservation of key residues in the NA active site.
(DOCX)

Table S12 Seroprevalence of IgG in Guatemalan bats to H17 rHA by ELISA.
(DOCX)

Text S1 Supporting Text. Methods, figure legends and tables.
(DOCX)
