# Supplementary figures and images for: New World Bats Harbor Diverse Influenza A Viruses
Source: PLoS Pathog. 2013 Oct 10;9(10):e1003657. doi: 10.1371/journal.ppat.1003657 (PMC3794996; doi:10.1371/journal.ppat.1003657)

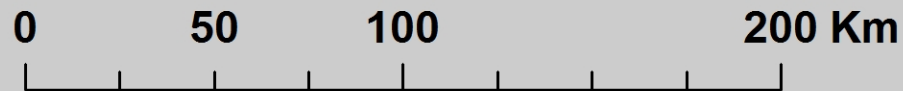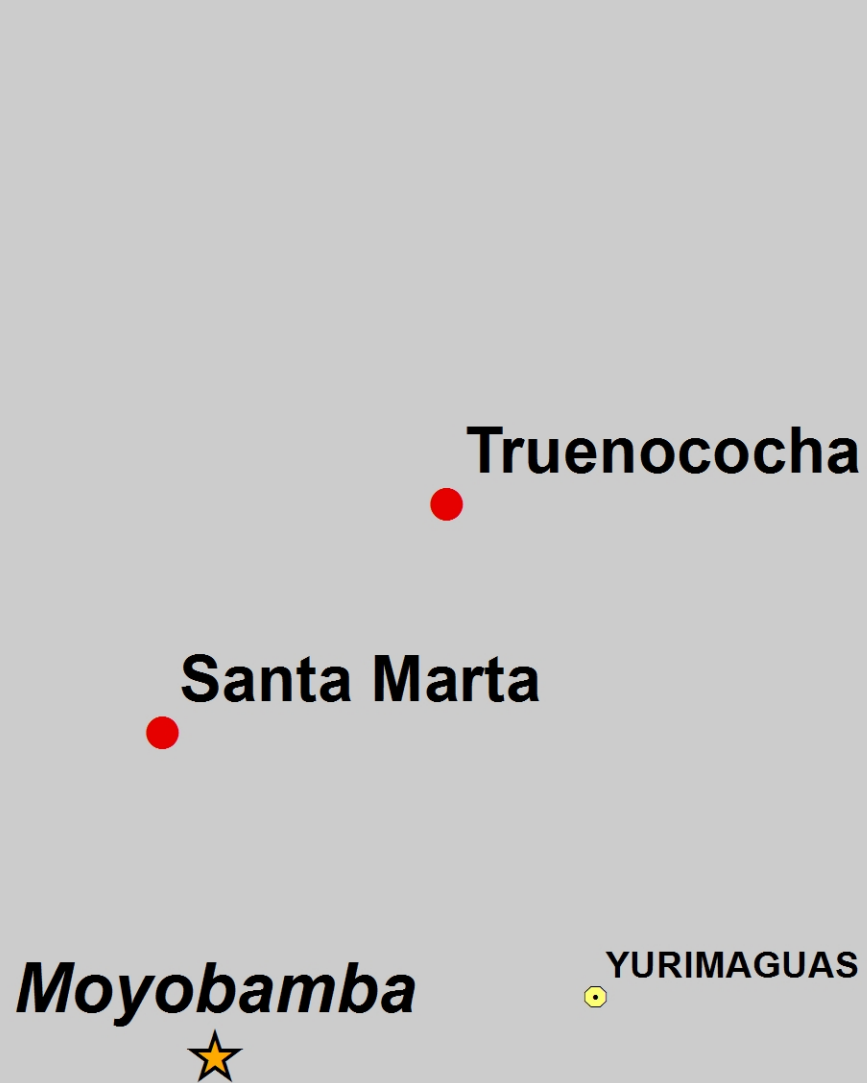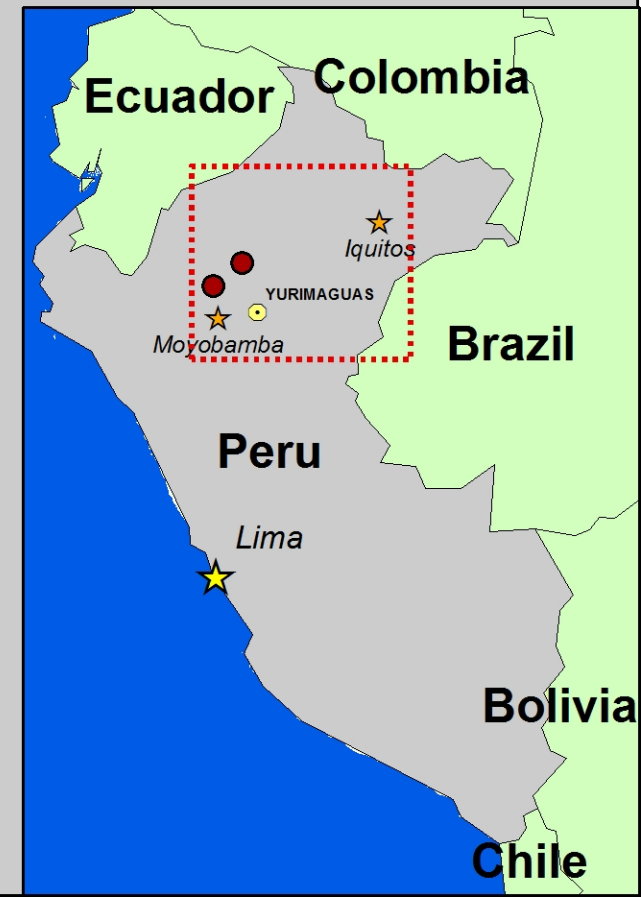

Supplement: Figure S1 — Geographic locations of bat-sampling sites in Peru. Bats were captured at Truenococha and Santa Marta in the Loreto District (the inset depicts this region within the Republic of Peru, see Table 1 and Table S1 for additional information). (PDF) [file ppat.1003657.s001.pdf]

Tong et al Fig. S2

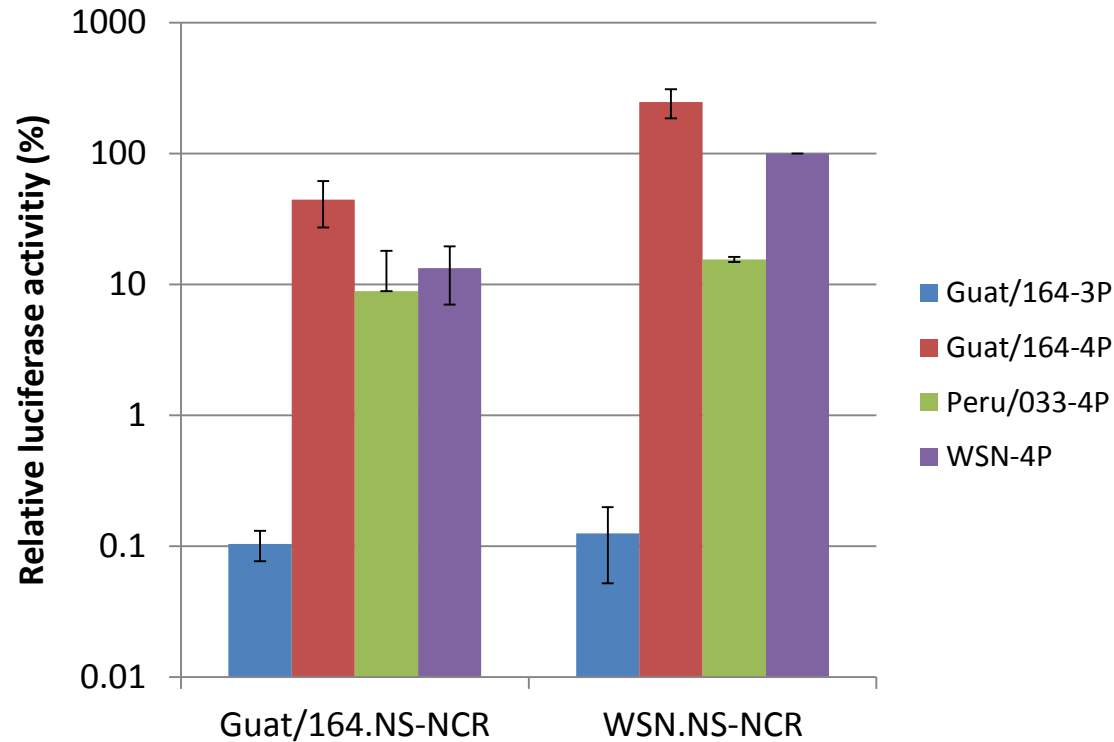

Supplement: Figure S2 — RNA-dependent RNA polymerase activity of A/bat/Peru/10 ribonucleoprotein (RNP) complex proteins (PB2, PB1, PA and NP). A549 human lung cells were transfected with pPol1-A/bat-NS.NCR-Renilla (Guat/164.NS-NCR) or pPol1-A/WSN-NS.NCR-Renilla (WSN.NS-NCR) and pSV40-Luc reporter plasmids, together with plasmids expressing PB2, PB1, PA and NP from either A/WSN/33 (WSN-4P) or A/bat/Peru/10 (Peru/033-4P) or A/bat/Guat/09 (Guat/164-4P) viruses or without the PB1 expression plasmid (Guat/164-3P). Values shown represent the activities of each RNP and reporter relative to that of WSN virus (100%) with the homologous reporter. Error bars indicate 95% confidence intervals. Experiments were performed three times independently. (PDF) [file ppat.1003657.s002.pdf]

**A**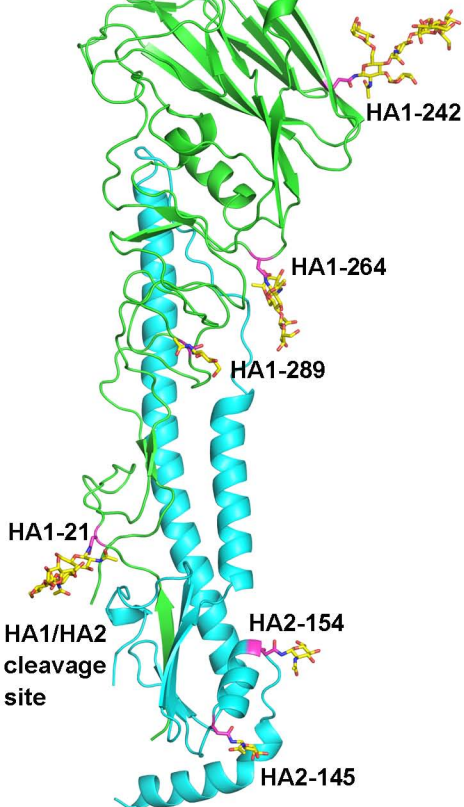**B**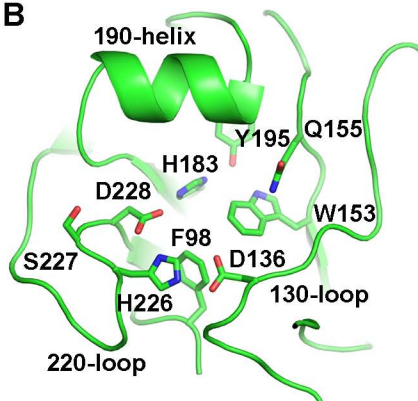**C**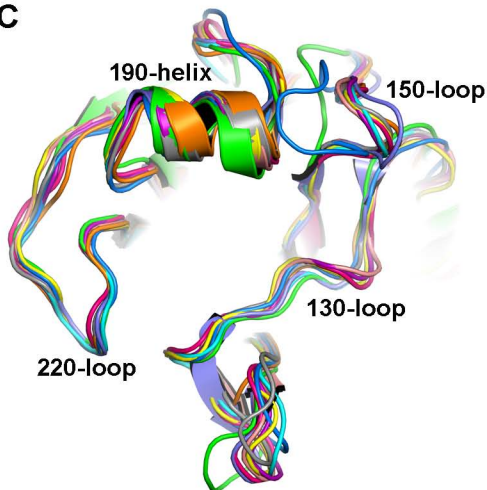

Supplement: Figure S3 — The structure of A/bat/Peru/10 HA in crystal 2. (A) One monomer is shown with the HA1 chain colored in green and the HA2 chain in cyan. The glycosylation positions are highlighted in magenta with the glycan in yellow. A/bat/Peru/10 HA has four potential N-linked glycosylation sites in HA1 (Asn21, Asn242, Asn264, Asn289) and a further two in HA2 (Asn145 and Asn154). While position 21 is close to the HA1/HA2 cleavage site, position 242 is closer to the putative receptor binding pocket. Positions 264 and 289 are close together in the middle of the molecule around the vestigial esterase domain in HAs from other influenza A viruses. In the HA2, positions 145 and 154 are near the membrane-anchoring region. Asn154 is conserved in all HAs, while Asn145 is only found in HA sequences from three other group 1 subtypes (H13, H16 and H17). From these structures, interpretable electron density for one or two N-acetyl glucosamines was observed at all six of these putative glycosylation sites. However, due to crystal packing, density for the carbohydrate at Asn242 was well defined in all three monomers and could be visualized up to the first three mannoses of the glycan. (B) The putative receptor binding site with the three structural elements, the 130-loop, 220-loop and the 190-helix. The putative binding site residues are shown in sticks. (C) Superposition of receptor binding site region of A/bat/Peru/10 H18 (in green), 1918 H1 (in salmon, PDB code 1RD8), 2009 H1 (in purple, 3M6S), swine H1 (in pink, 4F3Z), human H2 (in grey, 2WR7), human H5 (in yellow, 2FK0), swine H9 (in orange, 1JSD), human H3 (in cyan, 2HMG), human H7 (in marine, 4DJ6) and mallard H14 (in slate, 3EYJ). (PDF) [file ppat.1003657.s003.pdf]

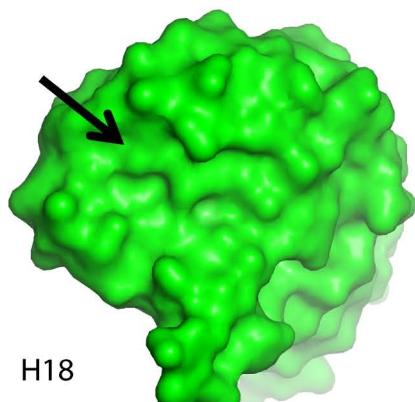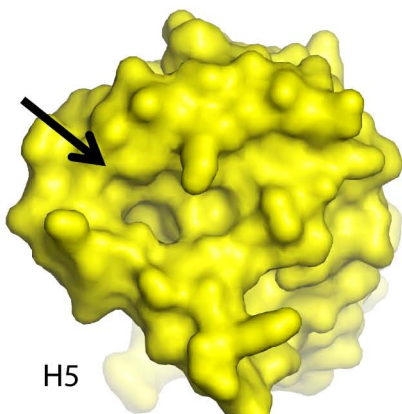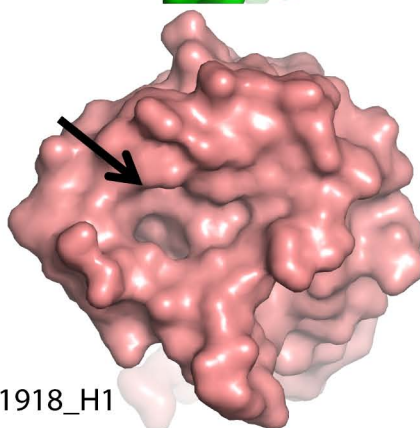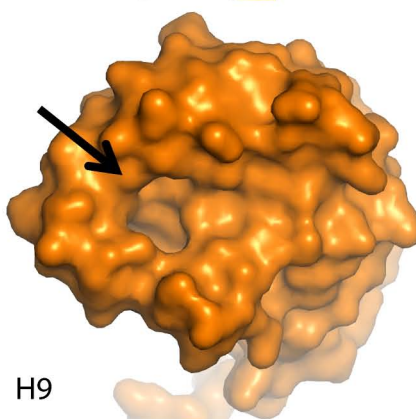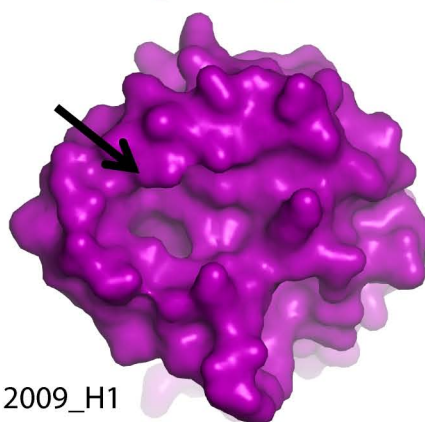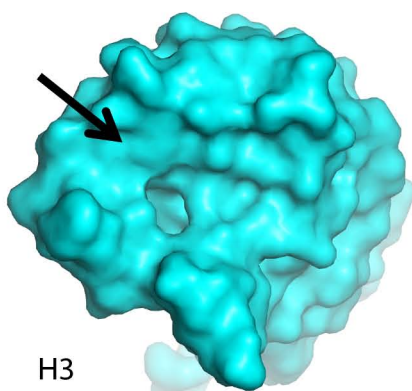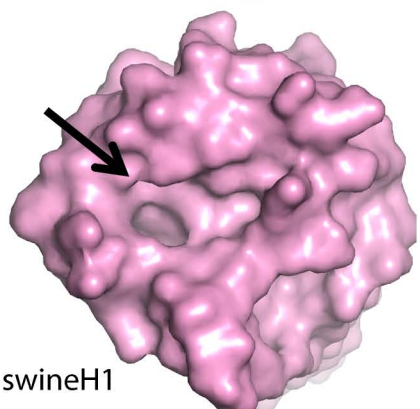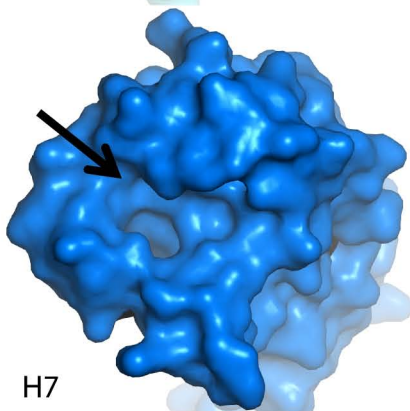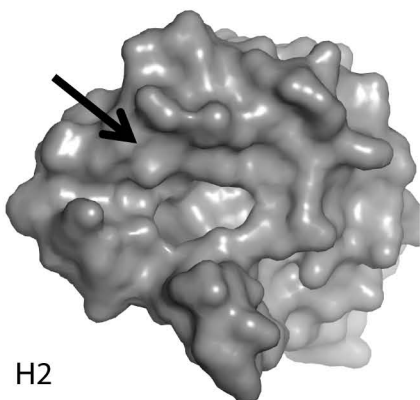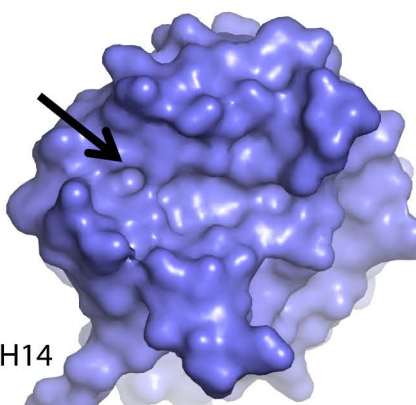

Supplement: Figure S4 — Comparative surface representation of the receptor binding sites of bat and non-bat HAs. A/bat/Peru/10 H18 HA (in green), 1918 H1 HA (salmon, PDB code 1RD8), 2009 H1 HA (purple, 3M6S), swine H1 HA (pink, 4F3Z), H2 HA (grey, 2WR7), H5 HA (yellow, 2FK0), H9 (orange, 1JSD), H3 HA (cyan, 2HMG), H7 HA (marine, 4DJ6) and H14 HA (slate, 2EYJ), with arrows indicating the receptor binding sites in other HAs. (PDF) [file ppat.1003657.s004.pdf]

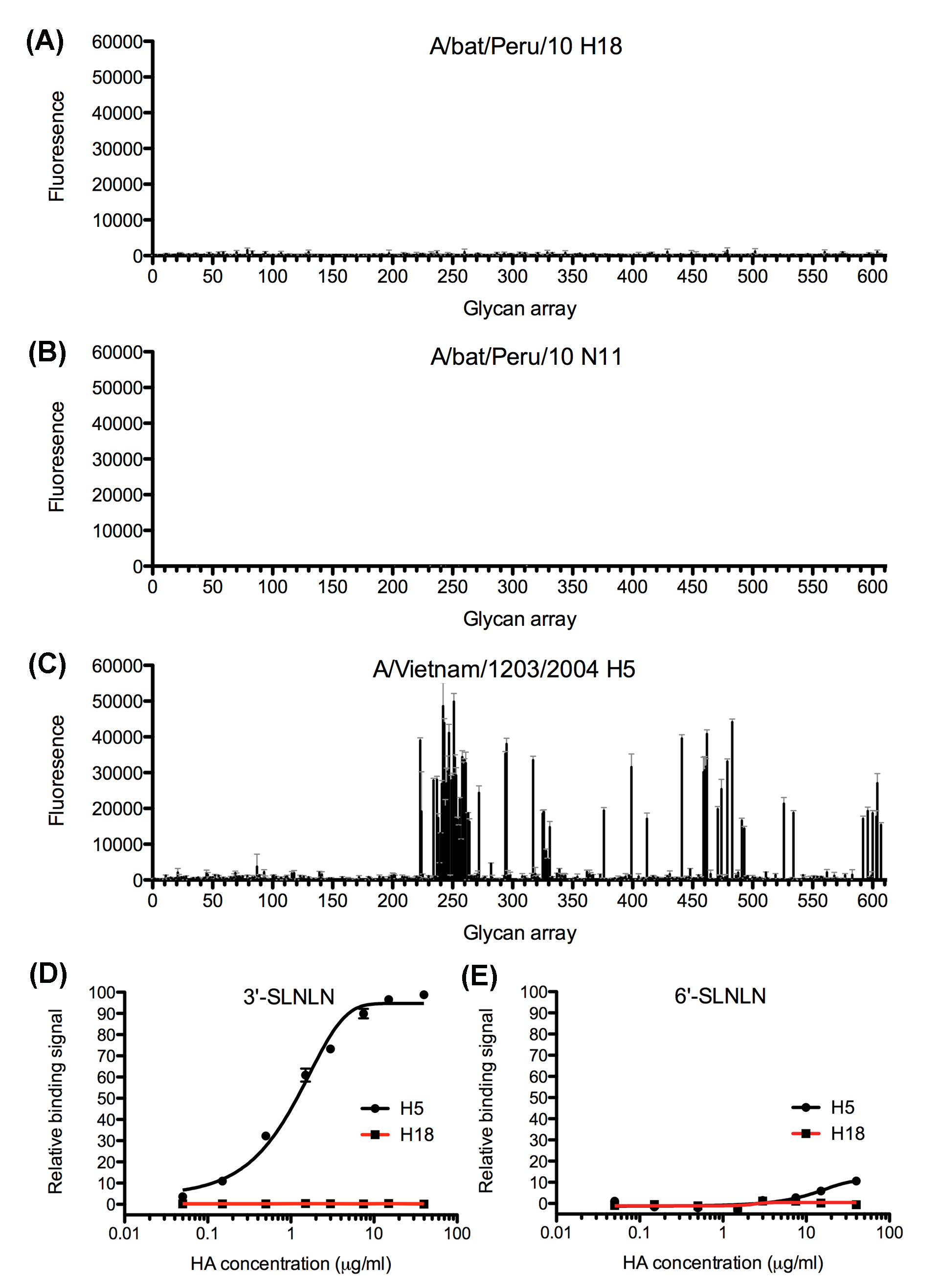

Supplement: Figure S5 — Glycan binding analysis of A/bat/Peru/10 HA and NAL. (A to C) Glycan microarray analysis of A/bat/Peru/10 HA (A) and NAL (B), and control protein A/Vietnam/1203/2004 H5 HA (C) was performed on the CFG glycan microarray v5.1, which contains 610 mammalian glycans. Binding signals (black bars) are shown in relative fluorescence units (RFU). The H5 HA showed good binding avidity to α2-3 glycans, but A/bat/Peru/10 HA and NAL exhibit no specific binding to any glycans on the array, including natural sialosides with α2-3, α2-6, α2-8 and mixed linkages, and other glycans that might exist in mammals (see Tables. S9 and S10). (D to E) ELISA-based plate assay of A/bat/Peru/10 HA and control A/Vietnam/1203/2004 H5 to 3′-SLNLN (D) and 6′-SLNLN (E). The H18 HA does not bind to either 3′-SLNLN or 6′-SLNLN in the experimental conditions, while the control H5 HA binds more strongly to 3′-SLNLN as expected. (TIF) [file ppat.1003657.s005.tif]

**A**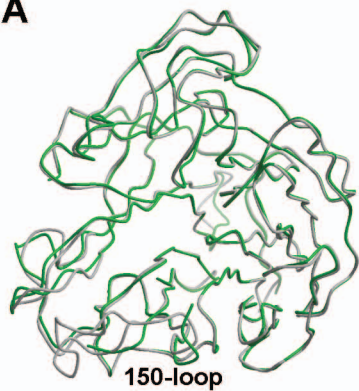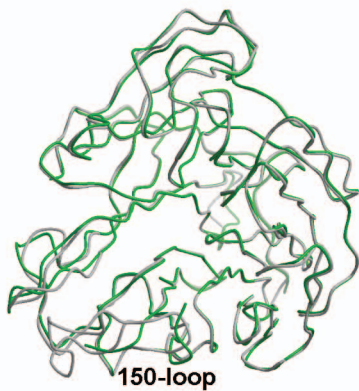**B**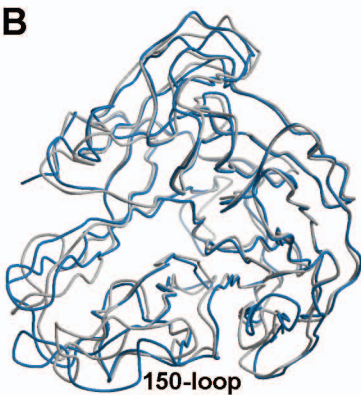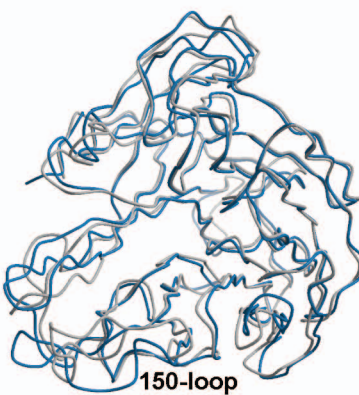**C**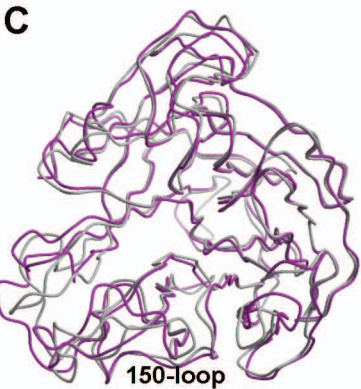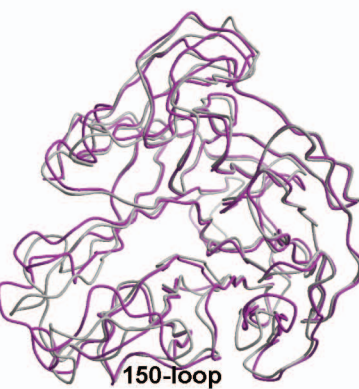**D**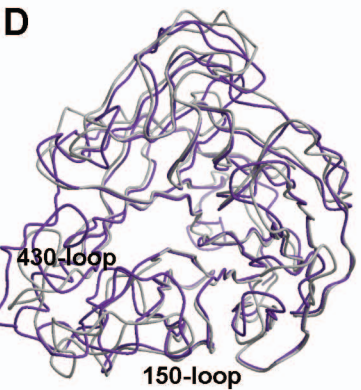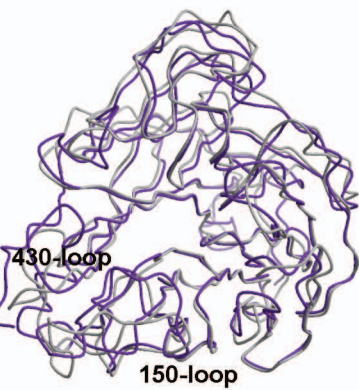

Supplement: Figure S6 — Stereo view of superimposed A/bat/Peru/10 NAL in crystal form 1 (in grey) with other NA structures. (A) Comparison with A/bat/Peru/10 NAL in crystal form 2 (in green) with Cα r.m.s.d. of 0.7 Å. The structures of A/bat/Peru/10 NAL in two crystal forms are the same except for the 150-loop, which is flexible in crystal form 2 and not modeled. (B) Comparison with GU09-164 NAL in crystal form 1 (in sky blue, PDB code 3GDI) with r.m.s.d. of 1.3 Å. The overall structures are very similar except for 150-loop which is closer to the putative active site in A/bat/Peru/10 N11 NAL, making the putative active site less open. (C) Comparison with GU09-164 NAL in crystal form 2 (in magenta, PDB code 3GEZ) with Cα r.m.s.d. of 1.2 Å. The overall structures are very similar except for 150-loop which is further from the putative active site in A/bat/Peru/10 N11 NAL, making the putative active site more open. (D) Comparison with 1918 N1 NA (in purple, PDB code 3BEQ) with Cα r.m.s.d. of 1.7 Å. As large conformational changes are observed in the 110-loop, 150-loop and 430-loop, as well as C-terminus, these regions were excluded from the RMSD calculation. Compared to 1918 N1 NA, the A/bat/Peru/10 NAL putative active site in crystal form 1 adopts a much more open conformation. (PDF) [file ppat.1003657.s006.pdf]

## NA activity against 4MU-NANA

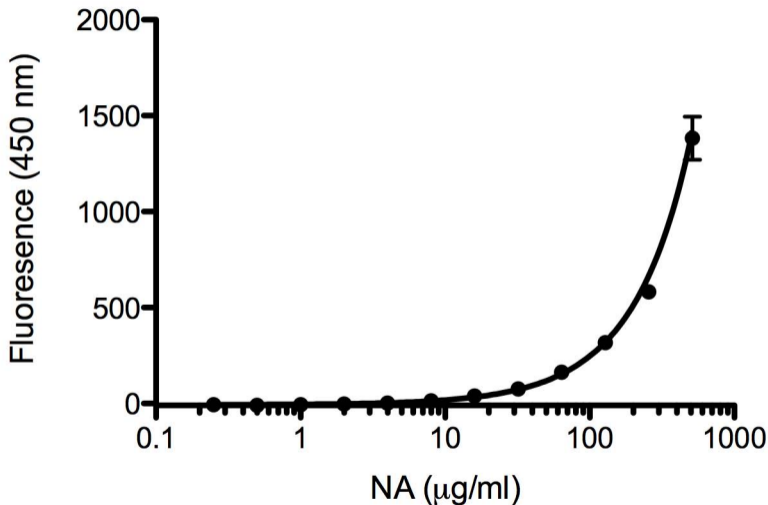

Supplement: Figure S7 — NA cleavage activity analysis of A/bat/Peru/10 NAL. Only extremely low sialic acid cleavage activity was observable with NAL concentrations as high as 100 µg/ml. (PDF) [file ppat.1003657.s007.pdf]

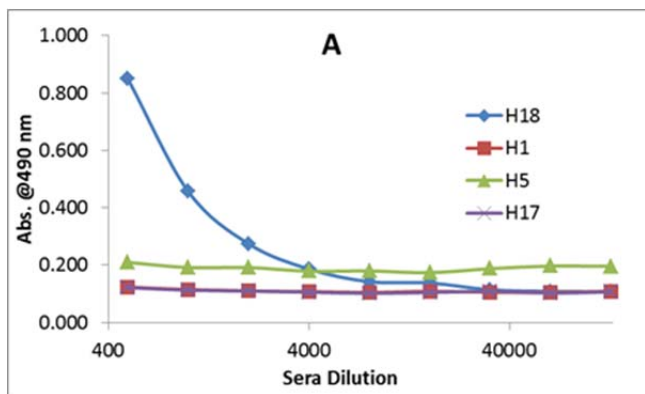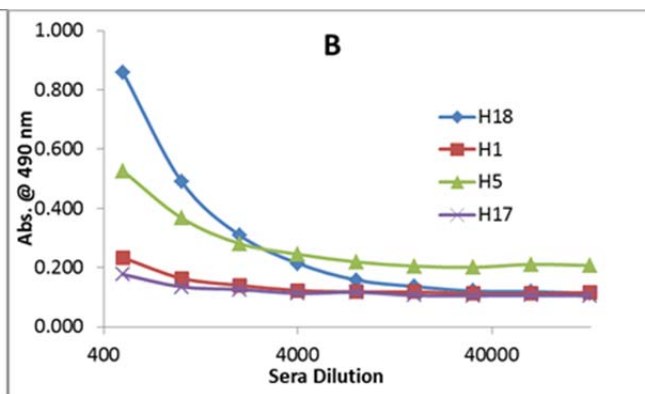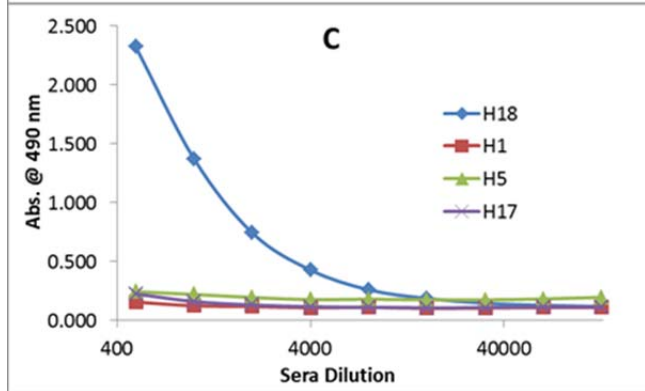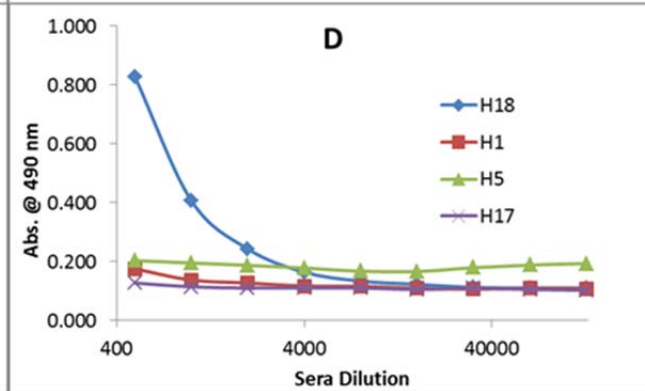

Supplement: Figure S8 — Detection of antibody to HA subtypes in bat sera by ELISA. Plates were coated with 1 ug/mL of H18 rHA antigen (homologous), or H17, H5,H1 rHA antigen (heterologous) to ascertain levels of cross reactivity. Serial log2 dilutions of sera were performed, starting at 1∶500, and an absorbance reading was taken at 490 nm for 0.1 seconds. Samples represented are (A) Peru 017, (B) Peru 019, (C) Peru 020, and (D) Peru 031. (PDF) [file ppat.1003657.s008.pdf]
