# Supplementary material for: New World Bats Harbor Diverse Influenza A Viruses
Source: PLoS Pathog. 2013 Oct 10;9(10):e1003657. doi: 10.1371/journal.ppat.1003657 (PMC3794996; doi:10.1371/journal.ppat.1003657)
Supplement: Table S1 — Nucleotide sequence identity between A/flat-faced bat/Peru/033/2010 (H18N11) and A/little yellow-shouldered bat/Guatemala/164/2009 (H17N10) genomes. (DOCX) [file ppat.1003657.s009.docx]

**Table S1. Nucleotide sequence identity between A/flat-faced bat/Peru/033/2010 (H18N11) and A/little yellow-shouldered bat/Guatemala/164/2009 (H17N10) genomes**

| A/bat/Peru/10 gene | % identity with A/bat/Guat/164/09 |
| --- | --- |
| PB2 | 76.5 |
| PB1 | 76.2 |
| PA | 78.3 |
| HA | 62.3 |
| NP | 78.9 |
| NA | 48.7 |
| M | 81.6 |
| NS | 78.9 |
