# Supplementary material for: New World Bats Harbor Diverse Influenza A Viruses
Source: PLoS Pathog. 2013 Oct 10;9(10):e1003657. doi: 10.1371/journal.ppat.1003657 (PMC3794996; doi:10.1371/journal.ppat.1003657)
Supplement: Table S2 — Mean amino acid identity between A/bat/Peru/10 H18 HA and influenza A subtypes H1–H17. (DOCX) [file ppat.1003657.s010.docx]

**Table S2. Mean amino acid identity between A/bat/Peru/10 H18 HA and influenza A subtypes H1-H17.**

|  |  |  | **Group 1** | | | | | | | | | | | | **Group 2** | | | | | | **All HAs (average)** |
| --- | --- | --- | --- | --- | --- | --- | --- | --- | --- | --- | --- | --- | --- | --- | --- | --- | --- | --- | --- | --- | --- |
|  | **HA** | **A/bat/Peru/10 H18 HA** | **H1** | **H2** | **H5** | **H6** | **H17** | **H11** | **H13** | **H16** | **H8** | **H9** | **H12** | **Average Group 1** | **H3** | **H4** | **H14** | **H7** | **H10** | **Average Group 2** |  |
|  |  |  |  |  |  |  |  |  |  |  |  |  |  |  |  |  |  |  |  |  |  |
| **Group 1** | **H1** | 50.2 |  |  |  |  |  |  |  |  |  |  |  |  |  |  |  |  |  |  |  |
|  | **H2** | 52.9 | 65.0 |  |  |  |  |  |  |  |  |  |  |  |  |  |  |  |  |  |  |
|  | **H5** | 54.5 | 63.0 | 75.9 |  |  |  |  |  |  |  |  |  |  |  |  |  |  |  |  |  |
|  | **H6** | 50.0 | 59.1 | 58.5 | 60.4 |  |  |  |  |  |  |  |  |  |  |  |  |  |  |  |  |
|  | **H17** | 60.2 | 51.8 | 54.4 | 53.6 | 52.3 |  |  |  |  |  |  |  |  |  |  |  |  |  |  |  |
|  | **H11** | 46.0 | 53.9 | 54.7 | 57.0 | 55.0 | 46.5 |  |  |  |  |  |  |  |  |  |  |  |  |  |  |
|  | **H13** | 45.5 | 50.2 | 50.3 | 51.2 | 51.2 | 45.6 | 59.1 |  |  |  |  |  |  |  |  |  |  |  |  |  |
|  | **H16** | 45.0 | 51.2 | 49.8 | 50.8 | 48.7 | 45.2 | 59.3 | 81.6 |  |  |  |  |  |  |  |  |  |  |  |  |
|  | **H8** | 46.1 | 51.0 | 48.5 | 50.3 | 53.7 | 48.3 | 48.9 | 48.1 | 49.5 |  |  |  |  |  |  |  |  |  |  |  |
|  | **H9** | 46.0 | 50.9 | 51.6 | 50.8 | 54.5 | 48.4 | 52.4 | 51.2 | 49.6 | 62.5 |  |  |  |  |  |  |  |  |  |  |
|  | **H12** | 43.8 | 47.4 | 48.9 | 48.8 | 51.2 | 45.5 | 48.9 | 49.3 | 49.6 | 64.3 | 65.3 |  |  |  |  |  |  |  |  |  |
|  | **Average Group 1** | 49.1 |  | | | | | | | | | | | 53.5 |  | | | | |  |  |
| **Group 2** | **H3** | 36.3 | 42.1 | 41.4 | 42.5 | 42.0 | 36.6 | 39.6 | 37.7 | 37.9 | 41.4 | 40.7 | 40.3 |  |  |  |  |  |  |  |  |
|  | **H4** | 37.6 | 42.3 | 42.5 | 41.8 | 42.2 | 37.3 | 41.2 | 40.8 | 40.4 | 44.6 | 43.2 | 42.5 |  | 64.1 |  |  |  |  |  |  |
|  | **H14** | 38.1 | 42.6 | 42.0 | 43.1 | 43.2 | 38.9 | 41.4 | 43.3 | 41.9 | 43.3 | 42.6 | 41.1 |  | 64.8 | 77.9 |  |  |  |  |  |
|  | **H7** | 39.5 | 43.2 | 41.5 | 42.2 | 41.7 | 38.3 | 41.1 | 39.4 | 38.4 | 40.0 | 39.3 | 41.0 |  | 46.9 | 47.2 | 48.1 |  |  |  |  |
|  | **H10** | 37.5 | 44.1 | 42.9 | 42.1 | 42.9 | 36.8 | 42.7 | 41.0 | 40.0 | 41.8 | 41.2 | 40.8 |  | 49.5 | 50.0 | 49.6 | 61.8 |  |  |  |
|  | **H15** | 40.1 | 43.4 | 41.4 | 40.9 | 42.3 | 37.5 | 41.9 | 40.0 | 38.6 | 40.4 | 39.7 | 40.9 |  | 47.1 | 48.2 | 49.7 | 80.0 | 65.2 |  |  |
|  | **Average Group 2** | 38.2 |  | | | | | | | | | | |  |  | | | | | 56.7 |  |
| **All HAs (average)** | | 45.3 |  | | | | | | | | | | | | | | | | | | 47.8 |
