# Supplementary material for: New World Bats Harbor Diverse Influenza A Viruses
Source: PLoS Pathog. 2013 Oct 10;9(10):e1003657. doi: 10.1371/journal.ppat.1003657 (PMC3794996; doi:10.1371/journal.ppat.1003657)
Supplement: Table S3 — Sequence comparison after sequence and structural alignment of HA1 (top) and HA2 (bottom) of A/bat/Peru/10 H18 HA with bat H17 HAs and non-bat HAs a. (DOCX) [file ppat.1003657.s011.docx]

**Table S3**. **Sequence comparison after sequence and structural alignment of HA1 (top) and HA2 (bottom)**

**of A/bat/Peru/10 H18 HA with bat H17 HAs and non-bat HAs ^a^.**

**________________________________________________________________**

**1 10 20 30 40 50 54**

**A/bat/Peru/10 H18 -------------MITILILVLP--IVVG----DQICIGYHSNNSTQTVNTLLESNVPV-TSSHSILEKEHNGLLCKL**

**GU09-164[H17N10] -----------MELIVLLILLNPYTFVLG----DRICIGYQANQNNQTVNTLLEQNVPV-TGAQEILETNHNGKLCSL**

**GU10-060[H17N10] -----------MELIILLILLNPYTFVLG----DRICIGYQANQNNQTVNTLLEQNVPV-TGAQEILETNHNGKLCSL**

**SC18 [H1N1] ------------MEARLLVLLCAFAATNA----DTICIGYHANNSTDTVDTVLEKNVTV-THSVNLLEDSHNGKLCKL**

**HK68[H3N2] -------MKTIIALSYIFCLALGQDLPGNDNSTATLCLGHHAVPNGTLVKTITDDQIEV-TNATELVQSSSTGKICNN**

**B/HK73 -------MKAIIVLLMVVTSN-----------ADRICTGITSSNSPHVVKTATQGEVNVTGVIPLTTTPTKSHFANLK**

**55 60 70 80 90 100 110 120**

**A/bat/Peru/10 H18 KGKAPLDL-----IDCSLPAWLMGNPKCDELLTASEWAYIKEDPEPENGICFPG-DF-DSLEDLILLVSNTDHFR-KE**

**GU09-164[H17N10] NGVPPLDL-----QSCTLAGWLLGNPNCDSLLEAEEWSYIKINESAPDDLCFPG-NF-ENLQDLLLEMSGVQNFT-KV**

**GU10-060[H17N10] NGVPPLDL-----QSCTLAGWLLGNPNCDNLLEAEEWSYIKINENAPDDLCFPG-NF-ENLQDLLLEMSGVQNFT-KV**

**SC18 [H1N1] KGIAPLQL-----GKCNIAGWLLGNPECDLLLTASSWSYIVETSNSENGTCYPG-DF-IDYEELREQLSSVSSFE-KF**

**HK68[H3N2] P-HRILDG-----IDCTLIDALLGDPHCDVFQN-ETWDLFVERSKAFSN-CYPY-DV-PDYASLRSLVASSGTLE-FI**

**B/HK73 G-TQTRGKLCPNCLNCTDLDVALGRPKCMG-TIPSAKASILHEVKPVTSGCFPIMHDRTKIRQLPNLLRGYENIRLSA**

**122 130 140 150 160 170 180 189**

**A/bat/Peru/10 H18 KIID-MTRFS--DVTTN-NVDSACPYDTNGASFYRNLNWVQQ--NKGKQ-LIFHYQNSEN--NPLLIIWGVHQTSNAA**

**GU09-164[H17N10] KLFN-PQSMT--GVTTN-NVDQTCPFE-GKPSFYRNLNWIQG--NSGLP-FNIEIKNPTS--NPLLLLWGIHNTKDAA**

**GU10-060[H17N10] KLFN-PQSMT--GVTTN-NVDQTCPFE-GKPSFYRNLNWIQG--NSGLP-FNIEIKNPTS--NPLLLLWGIHNTKDAA**

**SC18 [H1N1] EIFPKTSSWP--NHETTKGVTAACSYA-GASSFYRNLLWLTKKGSSYPK-LSKSYVNNKG--KEVLVLWGVHHPPTGT**

**HK68[H3N2] TEGF---TWT--GVTQN-GGSNACKRG-PGNGFFSRLNWLTKSGSTYPV-LNVTMPNNDN--FDKLYIWGVHHPSTNQ**

**B/HK73 RNVTNAETAPGGPYIV--GTSGSCPNVTNGNGFFATMAWAVPKNKTATNPLTVEVPYICTKGEDQITVWGFHSD-DET**

**190 200 210 220 230 240 250 260**

**A/bat/Peru/10 H18 EQNTYYGSQT-GSTTITIGEETNTYPLVISE-SSI---LNG-HSDRINYFWGVVNPNQNFSIVSTGNFIWPEYGYFFQ**

**GU09-164[H17N10] QQRNLYGNDY-SYTIFNFGEKSEEFRPEIGQ-RDE---VKA-HQDRIDYYWGSLPAQSTLRIESTGNLIAPEYGFYYK**

**GU10-060[H17N10] QQRNLYGNDY-SYTIFNFGEKSEEFRPDIGQ-RDE---IKA-HQDRIDYYWGSLPAQSTLRIESTGNLIAPEYGFYYK**

**SC18 [H1N1] DQQSLYQNAD-AYVSVGSSKYNRRFTPEIAA-RPK---VRD-QAGRMNYYWTLLEPGDTITFEATGNLIAPWYAFALN**

**HK68[H3N2] EQTSLYVQES-GRVTVSTRRSQQSIIPNIGS-RPW---VRG-QSSRISIYWTIVKPGDVLVINSNGNLIAPRGYFKMR**

**B/HK73 QMVKLYGDSKPQKFTSSANGVTTHYVSQIGGFPNQAEDEGLPQSGRIVVDYMVQKPGKTGTIAYQRGVLLPQKVWCAS**

**262 270 280 290 300 310 320 329**

**A/bat/Peru/10 H18 KTTNISGIIKSSEKISDCDTICQTK-IGAINSTL-PFQNIHQNAIGDCPKYVKAQELVLATGL--RNNP--IKETR**

**GU09-164[H17N10] RKEGKGGLMKSKLPISDCSTKCQTP-LGALNSTL-PFQNVHQQTIGNCPKYVKATSLMLATGL--RNNP--QMEGR**

**GU10-060[H17N10] RKEGKGGLMKSKLPISDCSTKCQTP-LGALNSTL-PFQNVHQQTIGNCPKYVKATSLMLATGL--RNNP--QMEGR**

**SC18 [H1N1] RG-SGSGIITSDAPVHDCNTKCQTP-HGAINSSL-PFQNIHPVTIGECPKYVRSTKLRMATGL--RNIP--SIQSR**

**HK68[H3N2] TG-K-SSIMSSDAPIDTCISECITP-NGSIPNDK-PFQNVNKITYGACPKYVKQNTLKLATGM--RNVP--EKQTR**

**B/HK73 GR-S-KVIKGSLPLIG-EAD-CLHEKYGGLNKSKPYYTGEHAKAIGNCPIWVKT--PLKLANGTKYRPPAKLLKER**

**1 10 20 30 40 50 60 70 78**

**A/bat/Peru/10 H18 GLFGAIAGFIEGGWQGLIDGWYGYHHQNSEGSGYAADKEATQKAVDAITTKVNNIIDKMNTQFESTAKEFNKIEMRIK**

**GU10-060[H17N10] GLFGAIAGFIEGGWQGMIDGWYGYHHENQEGSGYAADKEATQKAVDAITNKVNSIIDKMNSQFESNIKEFNRLELRIQ**

**GU09-164[H17N10] GLFGAIAGFIEGGWQGMIDGWYGYHHENQEGSGYAADKEATQKAVDAITNKVNSIIDKMNSQFESNIKEFNRLELRIQ**

**SC18 [H1N1] GLFGAIAGFIEGGWTGMIDGWYGYHHQNEQGSGYAADQKSTQNAIDGITNKVNSVIEKMNTQFTAVGKEFNNLERRIE**

**HK68[H3N2] GLFGAIAGFIENGWEGMIDGWYGFRHQNSEGTGQAADLKSTQAAIDQINGKLNRVIEKTNEKFHQIEKEFSEVEGRIQ**

**B/HK73 GFFGAIAGFLEGGWEGMIAGWHGYTSHGAHGVAVAADLKSTQEAINKITKNLNSLSELEVKNLQRLSGAMDELHNEIL**

**defgabcdefgabcdefgabcdefgabcdefgabcdefgabcdefgabcdefgab**

**80 90 100 110 120 130 140 150 156**

**A/bat/Peru/10 H18 HLSDRVDDGFLDVWSYNAELLVLLENERTLDFHDANVNNLYQKVKVQLKDNAIDMGNGCFKILHKCNNTCMDDIKNGT**

**GU10-060[H17N10] HLSDRVDDALLDIWSYNTELLVLLENERTLDFHDANVKNLFEKVKAQLKDNAIDEGNGCFLLLHKCNNSCMDDIKNGT**

**GU09-164[H17N10] HLSDRVDDALLDIWSYNTELLVLLENERTLDFHDANVKNLFEKVKAQLKDNAIDEGNGCFLLLHKCNNSCMDDIKNGT**

**SC18 [H1N1] NLNKKVDDGFLDIWTYNAELLVLLENERTLDFHDSNVRNLYEKVKSQLKNNAKEIGNGCFEFYHKCDDACMESVRNGT**

**HK68[H3N2] DLEKYVEDTKIDLWSYNAELLVALENQHTIDLTDSEMNKLFEKTRRQLRENAEDMGNGCFKIYHKCDNACIESIRNGT**

**B/HK73 ELDEKVDDLRADTISSQIELAVLLSNEGIINSEDEHLLALERKLKKMLGPSAVDIGNGCFETKHKCNQTCLDRIAAGT**

**cdefgabcdefgabcdefgabcdefgabcdefgabcdefgabcdefgabcdefga**

**160 170 175**

**A/bat/Peru/10 H18 YNYYEYRKESHLEK-QKID**

**GU10-060[H17N10] YKYMDYREESHIEK-QKIDG**

**GU09-164[H17N10] YKYMDYREESHIEK-QKIDG**

**SC18 [H1N1] YDYPKYSEESKLNR-EEIDG**

**HK68[H3N2] YDHDVYRDEALNNR-FQIKG**

**B/HK73 FNAGEFSLPTFDSLNITAAS ________________________________________________________________**

^a^ Abbreviations: A/bat/Peru/10, A/flat-faced fruit bat/Peru/033/2010 (H18N11); GU10-060, A/little yellow-shouldered bat/Guatemala/060/2010 (H17N10); GU09-164, A/little yellow-shouldered bat/Guatemala/164/2009 (H17N10); SC18, A/South Carolina/1/18 (H1N1); HK68, A/Hong Kong/68 (H3N2); B/HK73, B/Hong Kong/8/73. Green indicates important residues around the receptor-binding site in all flu A HAs [[16](#_ENREF_16)]. The putative fusion peptide in A/bat/Peru/10 HA is colored red and highly conserved with all other HAs. The conservation of the heptad repeats of HA2 and their positions in the coil (a-g) are shown in yellow and cyan.
