# Supplementary material for: New World Bats Harbor Diverse Influenza A Viruses
Source: PLoS Pathog. 2013 Oct 10;9(10):e1003657. doi: 10.1371/journal.ppat.1003657 (PMC3794996; doi:10.1371/journal.ppat.1003657)
Supplement: Table S4 — Mean amino acid identity between the A/bat/Peru/10 N11 NAL and representative NAs of influenza A and B viruses. (DOCX) [file ppat.1003657.s012.docx]

**Table S4. Mean amino acid identity between the A/bat/Peru/10 N11 NAL and representative NAs of influenza A and B viruses.**

|  | **NA subtype** | **A/bat/Peru/10**  **N11** | **N1** | **N2** | **N3** | **N4** | **N5** | **N6** | **N7** | **N8** | **N9** | **N10** |
| --- | --- | --- | --- | --- | --- | --- | --- | --- | --- | --- | --- | --- |
|  | **N1** | 31.0 |  |  |  |  |  |  |  |  |  |  |
|  | **N2** | 25.0 | 45.2 |  |  |  |  |  |  |  |  |  |
|  | **N3** | 23.6 | 46.2 | 51.6 |  |  |  |  |  |  |  |  |
|  | **N4** | 31.3 | 69.7 | 45.5 | 46.5 |  |  |  |  |  |  |  |
|  | **N5** | 31.6 | 59.2 | 43.9 | 42.0 | 47.3 |  |  |  |  |  |  |
|  | **N6** | 26.1 | 46.2 | 49.5 | 47.9 | 43.6 | 46.8 |  |  |  |  |  |
|  | **N7** | 26.8 | 44.5 | 47.3 | 45.8 | 42.5 | 44.2 | 59.7 |  |  |  |  |
|  | **N8** | 32.4 | 56.8 | 46.8 | 43.5 | 59.2 | 72.2 | 47.1 | 43.4 |  |  |  |
|  | **N9** | 26.4 | 42.0 | 48.7 | 47.1 | 40.9 | 40.5 | 68.4 | 60.6 | 41.9 |  |  |
|  | **N10** | 42.0 | 27.8 | 23.6 | 26.8 | 28.7 | 28.5 | 22.6 | 26.6 | 27.0 | 22.3 |  |
|  | **Average influenza A** | 29.6 | 44.6 | | | | | | | | | |
|  | **Influenza B** | 26.3 | 33.6 | 30.4 | 30.9 | 33.6 | 34.5 | 31.7 | 31.5 | 34.0 | 32.1 | 23.7 |
|  | **Average influenza B** |  | 31.6 | | | | | | | | | |
