# Supplementary material for: New World Bats Harbor Diverse Influenza A Viruses
Source: PLoS Pathog. 2013 Oct 10;9(10):e1003657. doi: 10.1371/journal.ppat.1003657 (PMC3794996; doi:10.1371/journal.ppat.1003657)
Supplement: Table S5 — Sequence comparison of A/bat/Peru/10 N11 NAL with bat N10 NALs and other influenza NAsa. (DOCX) [file ppat.1003657.s013.docx]

**Table S5**. **Sequence comparison of A/bat/Peru/10 N11 NAL with bat N10 NALs and other influenza NAs^a^.**

**__________________________________________________________________________**

**1 10 20 30 40 50 54**

**A/bat/Peru/10 N11 MSFQTS-TCLLIVSLICGILTVCLQVLLPFILIWTNTEPNYSCECPAPNISLSCPNGTSVTY**

**GU10-060[H17N10] MSINGT-TCLLTLSLILNIVMIGLQVLMPFVLLWTNSPP------PEIYNSTSCCNGTFLNE**

**GU09-164[H17N10] MSINGT-TCLLTLSLILNVIMIGLQILMPFILLWTNSPP------PEISNSTSCCNGTFLNE**

**A/Tokyo/3/67[H2N2] MNPNQKIITIGSVSLTIATVCFLMQIAILVTTVTLHFK--------QHDCDSPASNQVMPCE**

**A/Brevig Mission/1/18[H1N1] MNPNQKIITIGSICMVVGIISLILQIGNIISIWVSHSIQ------TGNQNHPETCNQSIITY**

**B/Beijing/1/87 MLPSTI-QTLTLFLTSGGVLLSLYVSASLSYLLYSDIL-----------LKFSSKITAPTMT**

**55 60 70 80 90 100 110**

**A/bat/Peru/10 N11 DSK--------NITENSFYSSTTNYLSPVIATPLVLGENLCSINGWVPTYRGEGTTGKI---**

**GU10-060[H17N10] TNN--------NITN---ISQITNNFLKEEKFYWKARSQMCEVKGWVPTHRGFPWGPEL---**

**GU09-164[H17N10] TNN--------NITN---ISQITNNFLKEEKFYWRAKSQMCEVKGWVPTHRGFPWGPEL---**

**A/Tokyo/3/67[H2N2] PIIIERNITEIVYLN---NTTIEKEICPKVVEYRNWSKPQCQITGFAPFSKDNSIRLSA---**

**A/Brevig Mission/1/18[H1N1] ENNTWVNQTYVNISN---TNVVAGQDATS--VILTGNSSLCPISGWAIYSKDNGIRIGS---**

**B/Beijing/1/87 LDCANASNVQAVNRS---ATKEMTFLLPE-PEWTYP-RLSCQGSTFQKALLISPHRFGEARG**

**111 120 130 140 150 160 170**

**A/bat/Peru/10 N11 PDEQMLTRQNFVSCSDKECRRFFVSMGYGTTTNFADLIVSEQ---MNVYSVKLGDPPTPDKL**

**GU10-060[H17N10] PGDLILSRRAYVSCDLTSCFKFFIAYGLSANQHLLNTSMEWE---ESLYKTPIGSANTLSTS**

**GU09-164[H17N10] PGDLILSRRAYVSCDLTSCFKFFIAYGLSANQHLLNTSMEWE---ESLYKTPIGSASTLSTS**

**A/Tokyo/3/67[H2N2] GGDIWVTREPYVSCDPVKCYQFALGQGTTLDNKHSNDTVHDRIPHRTLLMNELGVPFHLG-T**

**A/Brevig Mission/1/18[H1N1] KGDVFVIREPFISCSHLECRTFFLTQGALLNDKHSNGTVKDRSPYRTLMSCPVGEAPSPYNS**

**B/Beijing/1/87 NSAPLIIRGPFIACGPKECKHFALTHYAAQPGGYYNGTREDRNKLRHLISVKLGKIPTVENS**

**173 180 190 200 210 220 230**

**A/bat/Peru/10 N11 KFEAVGWSASSCHDGFQWTVLSVAG---DGFVSILYGGIITDTIHPTNGGPLRTQASSCICN**

**GU10-060[H17N10] EMILPGRSSSACFDGLKWTVLVSNGRDRNSFIMIKYGEEITDTFSASRGGPLRLPNSECICV**

**GU09-164[H17N10] EMILPGRSSSACFDGLKWTVLVANGRDRNSFIMIKYGEEVTDTFSASRGGPLRLPNSECICI**

**A/Tokyo/3/67[H2N2] RQVCIAWSSSSCHDGKAWLHVCITGDDKNATASFIYDGRLVDSIGSWSQNILRTQESECVCI**

**A/Brevig Mission/1/18[H1N1] RFESVAWSASACHDGMGWLTIGISGPDNGAVAVLKYNGIITDTIKSWRNNILRTQESECACV**

**B/Beijing/1/87 IFHMAAWSGSACHDGREWTYIGVDGPDSNALIKIKYGEAYTDTYHSYANNILRTQESACNCI**

**234 240 250 260 270 280 290 294**

**A/bat/Peru/10 N11 DGTCYTIIADGTTYTASSHRLYRLVNGTSAGWKALDTTGFNFEFPTCYYT-SGKVKCTGTNL**

**GU10-060[H17N10] EGSCFVLVSDGPNVNQSVHRIYELQNGTVQRWKQLNTTGINFEYSTCYTI-NNLIKCTGTNL**

**GU09-164[H17N10] EGSCFVIVSDGPNVNQSVHRIYELQNGTVQRWKQLNTTGINFEYSTCYTI-NNLIKCTGTNL**

**A/Tokyo/3/67[H2N2] NGTCTVVMTDGSASGRADTRILFIEEGKIVHISPLAGSAQHVEECSCYPR-YPGVRCICRDN**

**A/Brevig Mission/1/18[H1N1] NGSCFTIMTDGPSNGQASYKILKIEKGKVTKSIELNAPNYHYEECSCYPD-TGKVMCVCRDN**

**B/Beijing/1/87 GGDCYLMITDGSASGISKCRFLKIREGRIIKEIFPTGRVEHTEECTCGFASNKTIECACRDN**

**295 300 310 320 330 340 350 356**

**A/bat/Peru/10 N11 WNDAKRPFLEFDQ-SFTYTFKEPCLGFLGDTPRGID--TTNYC-DKTTTEGEGGIQGFMIEG**

**GU10-060[H17N10] WNDAKRPLLRFTK-DLNYQIVEPCNGAPTDFPRGGL--TTPSC-KMAQEKGEGGIQGFILDE**

**GU09-164[H17N10] WNDAKRPLLRFTK-ELNYQIVEPCNGAPTDFPRGGL--TTPSC-KMAQEKGEGGIQGFILDE**

**A/Tokyo/3/67[H2N2] WKGSNRPVVDINMEDYSIDSSYVCSGLVGDTPRNDDRSSNSNCRNPNNERGTQGVKGWAFDN**

**A/Brevig Mission/1/18[H1N1] WHGSNRPWVSFDQ-NLDYQIGYICSGVFGDNPRPNDGTG--SC-GPVSSNGANGIKGFSFRY**

**B/Beijing/1/87 SYTAKRPFVKLNVETDTAEIRLMCTETYLDTPRPDDGSITGPC-ESNGDKGRGGIKGGFVHQ**

**357 360 370 380 390 400 410**

**A/bat/Peru/10 N11 S-----NSWIGRIINPGSKKGFEIYKFLGTLFS-VQ-TVGNRNYQLLS-NSTIGRSGLYQP-**

**GU10-060[H17N10] K-----PAWTSKTKTELSQNGFVLEQIPDGIES-EG-TVS-LSYELFS-NKRTGRSGFFQP-**

**GU09-164[H17N10] K-----PAWTSKTKAESSQNGFVLEQIPNGIES-EG-TVS-LSYELFS-NKRTGRSGFFQP-**

**A/Tokyo/3/67[H2N2] G----NDLWMGRTISKDLRSGYETFKVIGGWSTPNSKSQI-NRQVIVDSDNRSGYSGIFSV-**

**A/Brevig Mission/1/18[H1N1] D----NGVWIGRTKSTSSRSGFEMIWDPNGWTETDSSFS--VRQDIVAITDWSGYSGSFVQH**

**B/Beijing/1/87 RMASKIGRWYSRTMSKTERMGMELYVKYDGDPWTDSEALA-HSGVMVSMKEPGWYSFGFEI-**

**413 420 430 440 450 460 469**

**A/bat/Peru/10 N11 ---AYESR-DCQELCFWIEIAATTKAG----LSSNDLITFCGTGGSMPDVNWG----------**

**GU10-060[H17N10] ---KGDLISECQRVCFWLEIEDQTVGL----GMIQELSTFCGINSPVQNINWDS---------**

**GU09-164[H17N10] ---KGDLISGCQRICFWLEIEDQTVGL----GMIQELSTFCGINSPVQNINWDS---------**

**A/Tokyo/3/67[H2N2] ---E-GK--SCINRCFYVELIRGRKQETRVWWTSNSIVVFCGTSGTYGTGSWPDGANINFMPI**

**A/Brevig Mission/1/18[H1N1] PELT-GL--DCMRPCFWVELIRGQPKE-NTIWTSGSSISFCGVNSDTVGWSWPDGAELPFSIDK**

**B/Beijing/1/87 ---K-DK--KCDVPCIGIEMVHDGG---KKTWHSAATAIYCLMGSGQLLWDTVTGVDMAL---**

**______________________________________________________________**

^a^ Highly conserved sialic acid substrate-contacting residues (first-shell residues) in all flu A and B NAs are shown in purple, and green indicates largely conserved framework residues (second-shell residues) around active site in all the flu A and B NAs . Abbreviations: GU09-164, A/little yellow-shouldered bat/Guatemala/164/2009 (H17N10); GU10-060, A/little yellow-shouldered bat/Guatemala/060/2010 (H17N10).
