# Supplementary material for: New World Bats Harbor Diverse Influenza A Viruses
Source: PLoS Pathog. 2013 Oct 10;9(10):e1003657. doi: 10.1371/journal.ppat.1003657 (PMC3794996; doi:10.1371/journal.ppat.1003657)
Supplement: Table S6 — Data collection and refinement statistics of A/bat/Peru/10 HA and A/bat/Peru/10 NAL. (DOCX) [file ppat.1003657.s014.docx]

**Table S6**. **Data collection and refinement statistics of A/bat/Peru/10 HA and A/bat/Peru/10 NAL.**

| Data set | A/bat/Peru/10 HA crystal 1 | A/bat/Peru/10 HA crystal 2 | A/bat/Peru/10 NAL crystal form 1 | A/bat/Peru/10 NAL crystal form 2 |
| --- | --- | --- | --- | --- |
| Space group | I422 | I422 | I222 | P6_1_ |
| Unit cell (Å) | *a* = *b* = 238.9,  *c* = 160.8 | *a* = *b* = 239.1,  *c* = 161.2 | *a* = 123.4, b = 164.4,  *c* = 214.9 | *a* = b = 181.3,  *c* = 136.6 |
| Resolution (Å) ^a^ | 50.0-2.15 (2.23-2.15) | 50.0-2.24 (2.32-2.24) | 50.0-3.00 (3.11-3.00) | 50.0-2.68 (2.73-2.68) |
| X-ray source | APS 23ID-D | SSRL 11-1 | APS 22ID | SSRL 12-2 |
| Unique reflections | 121,108 | 111,081 | 41,701 | 70,321 |
| Redundancy ^a^ | 13.3 (6.4) | 6.5 (6.3) | 2.9 (2.5) | 3.2 (3.3) |
| Average I/σ(I) ^a^ | 31.5 (1.9) | 12.4 (2.4) | 9.1 (1.4) | 14.5 (1.0) |
| Completeness ^a^ | 97.1 (79.7) | 99.8 (98.8) | 94.5 (93.0) | 98.4 (99.6) |
| *R*_sym_^b^ | 0.11 (0.76) | 0.09 (0.59) | 0.10 (0.70) | 0.08 (0.69) |
| Monomers in a.u. | 3 | 3 | 4 | 4 |
| *V*_m_ (Å^3^/Da) | 3.2 | 3.4 | 3.4 | 3.1 |
| Reflections used in refinement | 121,054 | 111,074 | 39,587 | 70,273 |
| Refined residues | 1,476 | 1,482 | 1,452 | 1,417 |
| Refined waters | 745 | 1,034 | 0 | 117 |
| *R*_cryst_^c^ | 0.183 | 0.168 | 0.214 | 0.178 |
| *R*_free_^d^ | 0.220 | 0.200 | 0.260 | 0.220 |
| *B*-values (Å^2^)  Protein  Waters | 44.0  46.7 | 33.0  37.3 | 80.1  - | 73.6  58.5 |
| Wilson *B*-value (Å^2^)  Ramachandran plot (%)^e^ | 47.2  97.9, 0.2 | 34.4  98.0, 0.5 | 74.7  94.5, 0.5 | 79.8  95.2, 0.3 |
| rmsd bond (Å) | 0.008 | 0.007 | 0.014 | 0.009 |
| rmsd angle (deg.) | 1.1 | 1.1 | 1.4 | 1.3 |
| PDB codes | 4K3X | 4MC5 | 4MC7 | 4K3Y |

^a^ Parenthesis denote outer-shell statistics.

^b^ *R*_sym_ = ∑_h_∑_i_ |I_i_ (h) - <I(h)>| /∑_h_∑_i_ I_i_ (h), where < I(h)> is the average intensity of i symmetry-related observations of reflections with Bragg index h.

^c^ *R*_cryst_ = ∑_hkl_ |F_o_ - F_c_| / ∑_hkl_ |F_o_|, where F_o_ and F_c_ are the observed and calculated structure factors.

^d^ *R*_free_ was calculated as for *R*_cryst_, but on 5% of data excluded before refinement.

^e^ The values are percentage of residues in the favored and outliers regions analyzed by MolProbity.
