# Supplementary material for: New World Bats Harbor Diverse Influenza A Viruses
Source: PLoS Pathog. 2013 Oct 10;9(10):e1003657. doi: 10.1371/journal.ppat.1003657 (PMC3794996; doi:10.1371/journal.ppat.1003657)
Supplement: Table S7 — Comparison of Cα rmsd values (Å) of A/bat/Peru/10 HA (crystal 1) with other influenza A virus HAsa. (DOCX) [file ppat.1003657.s015.docx]

**Table S7.**  **Comparison of C_α_ rmsd values (Å) of A/bat/Peru/10 HA (crystal 1) with other influenza A virus HAs^a^.**

|  |  |  |  | Crystal 1  (PDB code AA) | | Crystal 2 (PDB code BB) | |
| --- | --- | --- | --- | --- | --- | --- | --- |
| Group | Subtype | Strain name | PDB code | HA1 | HA2 | HA1 | HA2 |
| 1 | H1 | A/South Carolina/1/1918 | 1RD8 | 1.4 | 1.2 | 1.4 | 1.1 |
| 1 | H1 | A/Darwin/2001/2009 | 3M6S | 1.3 | 0.7 | 1.4 | 0.8 |
| 1 | H1 | A/swine/Indiana/P12439/00 | 4F3Z | 1.5 | 0.9 | 1.5 | 0.9 |
| 1 | H2 | A/Singapore/ 1/57 | 2WR7 | 1.4 | 0.8 | 1.4 | 0.8 |
| 1 | H5 | A/Vietnam/1203/2004 | 2FK0 | 1.6 | 0.9 | 1.6 | 0.8 |
| 1 | H9 | A/swine/Hong Kong/9/98 | 1JSD | 1.9 | 1.0 | 1.9 | 1.0 |
| 2 | H3 | A/Hong Kong/19/1968 | 2HMG | 2.2 | 1.3 | 2.1 | 1.3 |
| 2 | H7 | A/Netherland/219/2003 | 4DJ6 | 2.1 | 1.3 | 2.2 | 1.3 |
| 2 | H14 | A/mallard/Astrakhan/263/1982 | 3EYJ | 2.0 | 1.3 | 2.0 | 1.3 |

^a^To analyze differences in the overall structure, C_α_ r.m.s.d. values (Å) were calculated between the HA1 and HA2 subdomains of different HAs superimposed by sequential and structural alignment onto the equivalent domains of A/bat/Peru/10 HA.
