# Supplementary material for: New World Bats Harbor Diverse Influenza A Viruses
Source: PLoS Pathog. 2013 Oct 10;9(10):e1003657. doi: 10.1371/journal.ppat.1003657 (PMC3794996; doi:10.1371/journal.ppat.1003657)
Supplement: Table S8 — Conservation of key residues in influenza A HA receptor-binding site a. (DOCX) [file ppat.1003657.s016.docx]

**Table S8**. **Conservation of key residues in influenza A HA receptor-binding site ^a^.**

| Amino acid | Residue number | | | | | | | | | | | |
| --- | --- | --- | --- | --- | --- | --- | --- | --- | --- | --- | --- | --- |
|  | 98 | 134 | 136 | 153 | 155 | 183 | 190 | 194 | 195 | 225 | 226 | 228 |
| Ala | 0 | 1 | 11 | 0 | 4 | 0 | 367 | 0 | 0 | 4 | 2 | 2 |
| Cys | 4 | 0 | 0 | 2 | 0 | 0 | 0 | 0 | 1 | 1 | 0 | 0 |
| Asp | 1 | 0 | 3 | 0 | 0 | 1 | 6,497 | 0 | 0 | 4,514 | 0 | 3 |
| Glu | 0 | 0 | 0 | 0 | 1 | 0 | 5,578 | 0 | 0 | 210 | 0 | 2 |
| Phe | 13 | 0 | 0 | 0 | 0 | 0 | 0 | 3 | 1 | 0 | 0 | 0 |
| Gly | 0 | 13,265 | 2 | 0 | 4 | 0 | 19 | 0 | 0 | 7,363 | 0 | 10,482 |
| His | 2 | 0 | 0 | 0 | 878 | 12,789 | 1 | 0 | 5 | 1 | 5 | 0 |
| Ile | 0 | 0 | 1 | 0 | 2,696 | 0 | 2 | 301 | 0 | 0 | 1,185 | 0 |
| Lys | 0 | 0 | 0 | 0 | 1 | 0 | 2 | 0 | 0 | 5 | 2 | 0 |
| Leu | 0 | 0 | 0 | 0 | 542 | 10 | 7 | 12,922 | 0 | 0 | 922 | 0 |
| Met | 0 | 0 | 0 | 1 | 0 | 0 | 3 | 0 | 0 | 0 | 5 | 0 |
| Asn | 0 | 3 | 0 | 0 | 0 | 464 | 309 | 0 | 1 | 968 | 0 | 0 |
| Pro | 0 | 0 | 3 | 0 | 1 | 2 | 0 | 16 | 0 | 0 | 3 | 0 |
| Gln | 0 | 0 | 0 | 0 | 3 | 1 | 5 | 0 | 0 | 0 | 10,029 | 0 |
| Arg | 0 | 1 | 1 | 3 | 0 | 3 | 0 | 1 | 0 | 3 | 60 | 9 |
| Ser | 0 | 1 | 8,104 | 1 | 4 | 0 | 1 | 0 | 1 | 6 | 0 | 2,646 |
| Thr | 0 | 0 | 5,150 | 0 | 5,124 | 0 | 121 | 1 | 0 | 3 | 0 | 0 |
| Val | 0 | 2 | 0 | 0 | 3,698 | 0 | 231 | 22 | 0 | 1 | 919 | 1 |
| Trp | 0 | 2 | 0 | 13,268 | 0 | 0 | 0 | 0 | 0 | 0 | 0 | 1 |
| Tyr | 13,256 | 0 | 0 | 0 | 264 | 0 | 2 | 1 | 13,261 | 0 | 0 | 0 |
|  |  |  |  |  |  |  |  |  |  |  |  |  |
| H18 | Phe ^c^ | Asn ^b^ | Asp^b^ | Trp | Gln ^b^ | His | Glu | Tyr ^d^ | Tyr | Gly | His^c^ | Asp^b^ |

^a^ The incidence of an amino acid occurring at certain position is shown. A total of 13,282 (13,283 including A/bat/Peru/10 H18) full-length, non-redundant HA sequences from all influenza A viruses were available in the Influenza A Virus Resource at the NCBI in September 4, 2012. Red residues for H18 are consistent with the consensus sequences at that position in other influenza HAs.

^b^ Only in three bat influenza A virus HAs of A/bat/Peru/10 H18, GU09-164 H17 and GU10-060 H17.

^c^ Present in three bat HAs and a few other influenza A HAs.

^d^ Only in A/bat/Peru/10 H18
