# Supplementary material for: New World Bats Harbor Diverse Influenza A Viruses
Source: PLoS Pathog. 2013 Oct 10;9(10):e1003657. doi: 10.1371/journal.ppat.1003657 (PMC3794996; doi:10.1371/journal.ppat.1003657)
Supplement: Table S11 — Conservation of key residues in the NA active site. (DOCX) [file ppat.1003657.s019.docx]

**Table S11**. **Conservation of key residues in the NA active site.**

|  | Flu A NA (10,322)^a^ | | Flu B NA (702)^b^ |
| --- | --- | --- | --- |
| Residues no. | A/bat/Peru/10 N11 | Consensus^c^ | Consensus^c^ |
| 118 | R(10311) | R(10311) | R(702) |
| 119 | Q(2)^g^ | E(10288) | E(700) |
| 151 | E(36) | D(10031) | D(696) |
| 152 | Q(2)^g^ | R(10309) | R(701) |
| 156 | M(2)^g^ | R(10306) | R(702) |
| 178 | W(10,313) | W(10313) | W(702) |
| 179 | S(10,317) | S(10317) | S(702) |
| 198 | -^h^ | D(9514) | D(698) |
| 222 | P(3)^d^ | I(10244) | I(694) |
| 224 | R(10,298) | R(10298) | R(697) |
| 227 | A(1)^f^ | E(10299) | E(702) |
| 274 | N(4)^e^ | H(9999) | H(698) |
| 276 | E(10,304) | E(10304) | E(702) |
| 277 | F(1)^f^ | E(10308) | E(702) |
| 292 | T(4)^e^ | R(10306) | R(701) |
| 294 | L(3)^d^ | N(10295) | N(701) |
| 371 | K(3)^g^ | R(10314) | R(701) |
| 406 | R(3)^d^ | Y(10311) | Y(701) |
| 425 | E(10,318) | E(10318) | E(702) |

^a^ The incidence of an amino acid occurring at certain position is shown in parentheses. A total of 10,321 full-length, non-redundant NA sequences (10,322 to include A/bat/Peru/10 N11) from all influenza A viruses were available in the Influenza Virus Resource at the NCBI in September 4, 2012. Conserved residues are shown in red.

^b^ A total of 702 full-length, non-redundant NA sequences from all influenza B viruses were available in the Influenza Virus Resource at the NCBI in September 4, 2012.

^c^ Most common residue at position by simple majority across all NA sequences.

^d^ Only in three bat influenza A virus NALs of A/bat/Peru/10 N11, GU09-164 and GU10-060 N10s.

^e^ Present in three bat and one other influenza A virus NAs.

^f^ Only in A/bat/Peru/10 N11.

^g^ In A/bat/Peru/10 N11 and one or two other influenza A virus NAs.

^h^ Sequence deletion.
