# Supplementary material for: New World Bats Harbor Diverse Influenza A Viruses
Source: PLoS Pathog. 2013 Oct 10;9(10):e1003657. doi: 10.1371/journal.ppat.1003657 (PMC3794996; doi:10.1371/journal.ppat.1003657)
Supplement: Table S12 — Seroprevalence of IgG in Guatemalan bats to H17 rHA by ELISA. (DOCX) [file ppat.1003657.s020.docx]

**Table S12. Seroprevalence of IgG in Guatemalan bats to H17 rHA by ELISA**

| **Species sampled in 2009** | **ELISA +** | **Tested** |
| --- | --- | --- |
| *Artibeus jamaicensis* | 9 | 12 |
| *Artibeus lituratus* | 2 | 7 |
| *Carolia perspicillata* | 1 | 3 |
| *Centurio senex* | 0 | 1 |
| *Desmodus rotundus* | 9 | 41 |
| *Glossophaga soricina* | 2 | 6 |
| *Micronicterius nicrotis* | 0 | 3 |
| *Phyllostomus discolor* | 2 | 2 |
| *Pteronotus davyi* | 0 | 5 |
| *Sturnira lilium* | 13 | 21 |
| *Sturnida ludovici* | 0 | 1 |
| *Vampyressa pusilla* | 0 | 2 |
| **Species sampled in 2010** |  |  |
| *Artibeus jamaicensis* | 8 | 24 |
| *Artibeus lituratus* | 3 | 5 |
| *Artibeus phaeotis* | 1 | 1 |
| *Artibeus toltecus* | 0 | 1 |
| *Carollia perspicillata* | 2 | 8 |
| *Desmodus rotundus* | 5 | 26 |
| *Eptesicus fuscus* | 0 | 2 |
| *Glossophaga soricina* | 7 | 13 |
| *Macrophyllum macrophyllum* | 1 | 1 |
| *Molossus sinaloae* | 0 | 2 |
| *Myotis nigricans* | 0 | 2 |
| *Platyrrhinus helleri* | 0 | 10 |
| *Sturnira lilium* | 21 | 28 |
| *Uroderma bilobatum* | 0 | 1 |
| Totals | 86 | 228 |
