# Supplementary material for: New World Bats Harbor Diverse Influenza A Viruses
Source: PLoS Pathog. 2013 Oct 10;9(10):e1003657. doi: 10.1371/journal.ppat.1003657 (PMC3794996; doi:10.1371/journal.ppat.1003657)
Supplement: Text S1 — Supporting Text. Methods, figure legends and tables. (DOCX) [file ppat.1003657.s021.docx]

**New World bats harbor diverse influenza A viruses**

**Supporting Information**

Suxiang Tong^1^, Xueyong Zhu^5^_,_ Yan Li^1^, Mang Shi^8^, Jing Zhang^1^, Melissa Bourgeois^2^, Hua Yang^2^, Xianfeng Chen^1^, Sergio Recuenco^3^, Jorge Gomez^10^, Li-Mei Chen^2^, Adam Johnson^2^, Ying Tao^1^, Cyrille Dreyfus^5^, Wenli Yu^5^, Ryan McBride^6^, Paul J. Carney^2^, Amy T. Gilbert^3^, Jessie Chang^2^, Zhu Guo^2^, Charles T. Davis^2^, James C. Paulson^5,6^, James Stevens^2^, Charles E. Rupprecht^3,4^, Edward C. Holmes^8,9^, Ian A. Wilson^5,7^, Ruben O. Donis^2^

^1^Division of Viral Diseases, ^2^Influenza Division, ^3^Division of High Consequence Pathogens and Pathology, Centers for Disease Control and Prevention, Atlanta, GA 30333, USA. ^4^Global Alliance for Rabies Control. ^5^Department of Integrative Structural and Computational Biology, The Scripps Research Institute, La Jolla, CA 92037, USA. ^6^Department of Chemical Physiology, The Scripps Research Institute, La Jolla, CA 92037, USA. ^7^Skaggs Institute for Chemical Biology, The Scripps Research Institute, La Jolla, CA 92037, USA. ^8^Sydney Emerging Infections and Biosecurity Institute, School of Biological Sciences and Sydney Medical School, The University of Sydney, Sydney, NSW 2006, Australia. ^9^Fogarty International Center, National Institutes of Health, Bethesda, MD 20892, USA. ^10^Direccion General de Epidemiologıa, Ministerio de Salud - MINSA, Lima, Peru.

*Correspondence and requests for materials should be addressed to R. O. D. (rvd6@cdc.gov), S. T. (sot1@cdc.gov), J. S ([fwb4@cdc.gov](mailto:fwb4@cdc.gov)), E. C. H. (edward.holmes@sydney.edu.au), C. E. R. (charles_rupprecht@yahoo.com) and I.A.W. (wilson@scripps.edu).

**SUPPORTING INFORMATION**

**Supporting methods**

**Cloning, expression and purification of A/bat/Peru/10 H18 HA protein used for crystal 2.** The cDNA corresponding to the ectodomain of A/bat/Peru/10 HA (residues 15-513, equivalent to 11-329 of HA1 and 1-174 of HA2 in H3 numbering) was cloned into the baculovirus transfer vector, pAcGP67-A (BD Biosciences) in frame with an N-terminal gp67 signal peptide, a C-terminal thrombin cleavage site, a foldon trimerization sequence [[1](#_ENREF_1)], and a His_6_-tag to enable protein purification. Protein expressed from High Five™ cells was purified from the culture supernatant by metal affinity chromatography, subjected to thrombin cleavage and gel filtration chromatography. The purified trimeric H18 HA was subsequently buffer exchanged into 10 mM Tris-HCl, 50 mM NaCl, pH 8.0 and concentrated to 12.5 mg/ml for crystallization trials. The protein sample contained additional plasmid-encoded residues at both the N- (ADPG) and C-termini (SGRLVPR).

**Cloning, expression and purification of A/bat/Peru/10 N11 NAL proteins used for crystal form 2 and for evaluation of neuraminidase activity.** The ectodomain (residues 84-448, equivalent to 82-459 in N2 numbering) and ectodomain plus stalk region (residues 30-448, 37-459 in N2 numbering) of A/bat/Peru/10 NAL protein were expressed in a baculovirus system for structural and functional analyses. The cDNAs corresponding to the NAL ectodomain and ectodomain plus stalk region of the A/bat/Peru/10 NA were inserted into a baculovirus transfer vector, pFastbacHT-A (Invitrogen) with an N-terminal gp67 signal peptide, a thrombin cleavage site, and a His_6_-tag [[2](#_ENREF_2)]. The constructed plasmids were used to transform DH10bac competent bacterial cells by site-specific transposition (Tn-7 mediated) to form a recombinant Bacmid with beta-galactosidase blue-white receptor selection. The purified recombinant bacmids were used to transfect Sf9 insect cells for overexpression. The N11 NAL proteins were produced by infecting suspension cultures of Hi5 cells with recombinant baculovirus at an MOI of 5-10 and incubated at 28 ºC shaking at 110 RPM. After 72 hours, Hi5 cells were removed by centrifugation and supernatants containing secreted, soluble NALs were concentrated and buffer-exchanged into 20 mM Tris pH 8.0, 300 mM NaCl, 2.5 mM CaCl_2_, and further purified by metal affinity chromatography using Ni-nitrilotriacetic acid (NTA) resin (Qiagen). For crystal structure determination, the N11 NAL ectodomain was digested with thrombin to remove the His_6_-tag. The cleaved NAL ectodomain was purified further by size exclusion chromatography on a Hiload 16/90 Superdex 200 column (GE healthcare) in 20 mM Tris pH 8.0, 100 mM NaCl, 10 mM CaCl_2_ and 0.02% NaN_3_. For NA solution-based activity assay, the uncleaved ectodomain plus stalk region of the A/bat/Peru/10 NAL with His_6_-tag attached was concentrated after Ni-NTA purification, in 100 mM imidazole-malate pH 6.15, 150 mM NaCl, 10 mM CaCl_2_, 0.02% NaN_3_.

**Crystal structure determination of A/bat/Peru/10 H18 HA in crystal form 2.** Initial crystallization trials were set up using a Topaz^TM^ Free Interface Diffusion (FID) Crystallizer system (Fluidigm Corporation). Crystals were observed in conditions containing various molecular weights of PEG polymer. Following optimization, diffraction quality crystals for A/bat/Peru/10 HA were obtained at 20 °C using a modified method for microbath under oil [[3](#_ENREF_3)], by mixing the protein with reservoir solution containing 0.05 M CaCl_2_, 30% (w/v) PEG MME 550 and 0.1 M Bis-Tris buffer at pH 6.5. Crystals were flash-cooled at 100 K. Data were collected at the Stanford Synchrotron Radiation Lightsource (SSRL) beamline 11-1 at 100 K and processed with the DENZO-SCALEPACK suite [[4](#_ENREF_4)].

The structure of A/bat/Peru/10 HA was determined by molecular replacement with Phaser [[5](#_ENREF_5)] using the H2 HA structure from A/Singapore/1/1957 (PDB code 2WR7, 53% identity) as the MR search model. The MR solution identified a trimer of three HA monomers related by a non-crystallographic 3-fold with an estimated solvent content of 63.7% based on a Matthews’ coefficient (*Vm*) of 3.4 Å^3^/Da. The model was then “mutated” to the correct sequence and rebuilt by Phenix Autobuild [[6](#_ENREF_6)] and Coot [[7](#_ENREF_7)], and the structure was refined with Refmac5 [[8](#_ENREF_8)] and Phenix Refine [[9](#_ENREF_9)]. The final model was assessed using MolProbity [[10](#_ENREF_10)]. Statistics on data processing and refinement are presented in Table S6.

**Crystal structure determination of A/bat/Peru/10 N11 NAL in crystal form 2.** Crystallization experiments were set up using the sitting drop vapor diffusion method. The A/bat/Peru/10 NAL crystal in form 2 was obtained by mixing the NAL ectodomain monomer at 10 mg/ml in 20 mM Tris pH 8.0, 100 mM NaCl, 10 mM CaCl_2_ and 0.02 % NaN_3_ with 0.2 M K_2_SO_4_, and 18% (w/v) polyethylene glycol 3350 at 22 °C. The A/bat/Peru/10 NAL crystals were cryoprotected in mother liquor with addition of 6.4% (w/v) PEG 3350 before being flash-cooled at 100 K. Diffraction data were collected at beamline 12-2 at SSRL. Data for all crystals were integrated and scaled with HKL2000 [[4](#_ENREF_4)].

The A/bat/Peru/10 NAL structure was determined by molecular replacement using the program Phaser [[5](#_ENREF_5)] with the A/little yellow-shouldered bat/Guatemala/164/2009 (H17N10) N10 NAL structure (PDB code 4GDI) as the MR starting model. Initial rigid body refinement was performed in Refmac5 [[8](#_ENREF_8)] and simulated annealing and restrained refinement (including TLS refinement) were carried out in Phenix [[11](#_ENREF_11)]. Between rounds of refinements, model building was carried out with the program Coot [[7](#_ENREF_7)]. Final data processing and refinement statistics are represented in Table S6. The quality of the structure was analyzed using the JCSG validation suite ([www.jcsg.org](http://www.jcsg.org/)). All figures were generated with Bobscript [[12](#_ENREF_12)] except for Fig. 2C and D, which was generated with PyMol ([www.pymol.org](http://www.pymol.org/)).

**HA enzyme-linked immunosorbent assay (ELISA) based plate assay for glycan binding.** Protocols for ELISA-based glycan binding assay were as previously described [[13](#_ENREF_13)]. Briefly, 50 µl of 1.6 mM biotinylated glycans (NeuAc*α*2-6Gal*β*1-4GlcNAc*β*1-3Gal*β*1-4GlcNAc (6’-SLNLN) and NeuAc*α*2-3Gal*β*1-4GlcNAc*β*1-3Gal*β*1-4GlcNAc (3’-SLNLN), obtained from the Consortium for Functional Glycomics) were loaded onto a streptavidin-coated high binding capacity 384-well plate (Pierce) overnight at 4 ºC. The plate was washed with PBS buffer and incubated with HA-antibody complexes comprised of His-tagged HA protein, and primary antibody (mouse anti-Penta-His antibody, Qiagen) and secondary antibody (horseradish peroxidase (HRP)-conjugated goat anti-mouse IgG, Pierce) at a weight ratio of 4:2:1. After incubation for 2 hours at room temperature (~22 ºC) and extensive wash with PBST and PBS, HRP activity was measured using Amplex Red hydrogen peroxide/peroxidase assay kit (Invitrogen).

**HA glycan microarray receptor binding assay.** Protocols for microarray HA analysis were as previously described [[14](#_ENREF_14),[15](#_ENREF_15)]. Briefly, HA-antibody complexes were prepared by mixing 15 µg of recombinant HA, mouse anti-His Alexa Fluor 488 (Qiagen) and goat anti-mouse IgG Alexa Fluor 488 (Invitrogen) in a molar ratio of 4:2:1, respectively, in 20 mM Tris pH 8.0, 100 mM NaCl, and 0.02% NaN_3_ buffer. These prepared complexes were incubated on ice for 15 min, and 100 µL of the complex mixture was then added directly to the surface of the array and allowed to incubate for 1 hour at room temperature (~ 22 ºC) in a humidified chamber, protected from the light. After the initial incubation, HA-antibody solution was removed by pipetting the solution and washing 3 times with 100 µL 1x PBS + 0.05% Tween, pH 7.4, and, subsequently, by dipping 3 times in 1x PBS and then 3 times in distilled H_2_O. Washed slides were dried by centrifugation and scanned on a ProScanArray Express HT (PerkinElmer) confocal slide scanner for AlexaFluor488 setting. Image data were stored as a TIFF image and signal data was collected using Imagene (BioDiscovery) imaging software. The signal data were processed to determine averaged (mean signal minus mean background) values of 4 replicate spots on the array for each unique printed glycan. Two different arrays were analyzed. One is a custom sialoside array comprising 58 sialosides described previously [[14](#_ENREF_14)], and the other is glycan array version 5.1 provided by the CFG containing 610 glycans of diverse structure (Table S9). Results from the v5.1 glycan microarray are deposited to the CFG database (http://www.functionalglycomics.org/).

**REFERENCES**

1. Frank S, Kammerer RA, Mechling D, Schulthess T, Landwehr R, et al. (2001) Stabilization of short collagen-like triple helices by protein engineering. J Mol Biol 308: 1081-1089.

2. Xu X, Zhu X, Dwek RA, Stevens J, Wilson IA (2008) Structural characterization of the 1918 influenza virus H1N1 neuraminidase. J Virol 82: 10493-10501.

3. Chayen NE, Shaw-Steward PD, Blow DM (1992) Microbatch crystallization under oil -- a new technique allowing many small volume crystallization experiments J Cryst Growth 122: 176-180.

4. Otwinowski A, Minor W (1997) Processing of X-ray diffraction data collected in oscillation mode. Methods Enzymol 276: 307-326.

5. McCoy AJ, Grosse-Kunstleve RW, Storoni LC, Read RJ (2005) Likelihood-enhanced fast translation functions. Acta Crystallogr D Biol Crystallogr 61: 458-464.

6. Afonine PV, Grosse-Kunstleve RW, Echols N, Headd JJ, Moriarty NW, et al. (2012) Towards automated crystallographic structure refinement with phenix.refine. Acta Crystallogr D Biol Crystallogr 68: 352-367.

7. Emsley P, Cowtan K (2004) Coot: model-building tools for molecular graphics. Acta Crystallogr D Biol Crystallogr 60: 2126-2132.

8. Vagin AA, Steiner RA, Lebedev AA, Potterton L, McNicholas S, et al. (2004) REFMAC5 dictionary: organization of prior chemical knowledge and guidelines for its use. Acta Crystallogr D Biol Crystallogr 60: 2184-2195.

9. Terwilliger TC, Grosse-Kunstleve RW, Afonine PV, Moriarty NW, Zwart PH, et al. (2008) Iterative model building, structure refinement and density modification with the PHENIX AutoBuild wizard. Acta Crystallogr D Biol Crystallogr 64: 61-69.

10. Chen VB, Arendall WB, 3rd, Headd JJ, Keedy DA, Immormino RM, et al. (2010) MolProbity: all-atom structure validation for macromolecular crystallography. Acta Crystallogr D Biol Crystallogr 66: 12-21.

11. Adams PD, Afonine PV, Bunkoczi G, Chen VB, Davis IW, et al. (2010) PHENIX: a comprehensive Python-based system for macromolecular structure solution. Acta Crystallogr D Biol Crystallogr 66: 213-221.

12. Esnouf RM (1997) An extensively modified version of MolScript that includes greatly enhanced coloring capabilities. J Mol Graph Model 15: 132-134, 112-133.

13. Xu R, McBride R, Nycholat CM, Paulson JC, Wilson IA (2012) Structural characterization of the hemagglutinin receptor specificity from the 2009 H1N1 influenza pandemic. J Virol 86: 982-990.

14. Blixt O, Head S, Mondala T, Scanlan C, Huflejt ME, et al. (2004) Printed covalent glycan array for ligand profiling of diverse glycan binding proteins. Proc Natl Acad Sci U S A 101: 17033-17038.

15. Zhu X, McBride R, Nycholat CM, Yu W, Paulson JC, et al. (2012) Influenza virus neuraminidases with reduced enzymatic activity that avidly bind sialic Acid receptors. J Virol 86: 13371-13383.

16. Skehel JJ, Wiley DC (2000) Receptor binding and membrane fusion in virus entry: the influenza hemagglutinin. Annu Rev Biochem 69: 531-569.

**SUPPORTING FIGURES**

**Fig. S1.** Geographic locations of bat-sampling sites in Peru. Bats were captured at Truenococha and Santa Marta in the Loreto District (the inset depicts this region within the Republic of Peru, see Table 1 and Table S1 for additional information).

**Fig. S2**. RNA-dependent RNA polymerase activity of A/bat/Peru/10 ribonucleoprotein (RNP) complex proteins (PB2, PB1, PA and NP). A549 human lung cells were transfected with pPol1-A/bat-NS.NCR-Renilla (Guat/164.NS-NCR) or pPol1-A/WSN-NS.NCR-Renilla (WSN.NS-NCR) and pSV40-Luc reporter plasmids, together with plasmids expressing PB2, PB1, PA and NP from either A/WSN/33 (WSN-4P) or A/bat/Peru/10 (Peru/033-4P) or A/bat/Guat/09 (Guat/164-4P) viruses or without the PB1 expression plasmid (Guat/164-3P). Values shown represent the activities of each RNP and reporter relative to that of WSN virus (100%) with the homologous reporter. Error bars indicate 95% confidence intervals. Experiments were performed three times independently.

**Fig. S3**. The structure of A/bat/Peru/10 HA in crystal 2. (A) One monomer is shown with the HA1 chain colored in green and the HA2 chain in cyan. The glycosylation positions are highlighted in magenta with the glycan in yellow. A/bat/Peru/10 HA has four potential N-linked glycosylation sites in HA1 (Asn21, Asn242, Asn264, Asn289) and a further two in HA2 (Asn145 and Asn154). While position 21 is close to the HA1/HA2 cleavage site, position 242 is closer to the putative receptor binding pocket. Positions 264 and 289 are close together in the middle of the molecule around the vestigial esterase domain in HAs from other influenza A viruses. In the HA2, positions 145 and 154 are near the membrane-anchoring region. Asn154 is conserved in all HAs, while Asn145 is only found in HA sequences from three other group 1 subtypes (H13, H16 and H17). From these structures, interpretable electron density for one or two N-acetyl glucosamines was observed at all six of these putative glycosylation sites. However, due to crystal packing, density for the carbohydrate at Asn242 was well defined in all three monomers and could be visualized up to the first three mannoses of the glycan. **(**B) The putative receptor binding site with the three structural elements, the 130-loop, 220-loop and the 190-helix. The putative binding site residues are shown in sticks. (C) Superposition of receptor binding site region of A/bat/Peru/10 H18 (in green), 1918 H1 (in salmon, PDB code 1RD8), 2009 H1 (in purple, 3M6S), swine H1 (in pink, 4F3Z), human H2 (in grey, 2WR7), human H5 (in yellow, 2FK0), swine H9 (in orange, 1JSD), human H3 (in cyan, 2HMG), human H7 (in marine, 4DJ6) and mallard H14 (in slate, 3EYJ).

**Fig. S4.** Comparative surface representation of the receptor binding sites of bat and non-bat HAs. A/bat/Peru/10 H18 HA (in green), 1918 H1 HA (salmon, PDB code 1RD8), 2009 H1 HA (purple, 3M6S), swine H1 HA (pink, 4F3Z), H2 HA (grey, 2WR7), H5 HA (yellow, 2FK0), H9 (orange, 1JSD), H3 HA (cyan, 2HMG), H7 HA (marine, 4DJ6) and H14 HA (slate, 2EYJ), with arrows indicating the receptor binding sites in other HAs.

**Fig. S5.** Glycan binding analysis of A/bat/Peru/10 HA and NAL. (A to C) Glycan microarray analysis of A/bat/Peru/10 HA (A) and NAL (B), and control protein A/Vietnam/1203/2004 H5 HA (C) was performed on the CFG glycan microarray v5.1, which contains 610 mammalian glycans. Binding signals (black bars) are shown in relative fluorescence units (RFU). The H5 HA showed good binding avidity to *α*2-3 glycans, but A/bat/Peru/10 HA and NAL exhibit no specific binding to any glycans on the array, including natural sialosides with *α*2-3, *α*2-6, *α*2-8 and mixed linkages, and other glycans that might exist in mammals (see Tables. S9 and S10). (D to E) ELISA-based plate assay of A/bat/Peru/10 HA and control A/Vietnam/1203/2004 H5 to 3’-SLNLN (D) and 6’-SLNLN (E). The H18 HA does not bind to either 3’-SLNLN or 6’-SLNLN in the experimental conditions, while the control H5 HA binds more strongly to 3’-SLNLN as expected.

**Fig. S6.** Stereo view of superimposed A/bat/Peru/10 NAL in crystal form 1 (in grey) with other NA structures. (A) Comparison with A/bat/Peru/10 NAL in crystal form 2 (in green) with C_α_ r.m.s.d. of 0.7 Å. The structures of A/bat/Peru/10 NAL in two crystal forms are the same except for the 150-loop, which is flexible in crystal form 2 and not modeled. (B) Comparison with GU09-164 NAL in crystal form 1 (in sky blue, PDB code 3GDI) with r.m.s.d. of 1.3 Å. The overall structures are very similar except for 150-loop which is closer to the putative active site in A/bat/Peru/10 N11 NAL, making the putative active site less open. (C) Comparison with GU09-164 NAL in crystal form 2 (in magenta, PDB code 3GEZ) with C_α_ r.m.s.d. of 1.2 Å. The overall structures are very similar except for 150-loop which is further from the putative active site in A/bat/Peru/10 N11 NAL, making the putative active site more open. (D) Comparison with 1918 N1 NA (in purple, PDB code 3BEQ) with C_α_ r.m.s.d. of 1.7 Å. As large conformational changes are observed in the 110-loop, 150-loop and 430-loop, as well as C-terminus, these regions were excluded from the RMSD calculation. Compared to 1918 N1 NA, the A/bat/Peru/10 NAL putative active site in crystal form 1 adopts a much more open conformation.

**Fig. S7.** NA cleavage activity analysis of A/bat/Peru/10 NAL. Only extremely low sialic acid cleavage activity was observable with NAL concentrations as high as 100 µg/ml.

**Fig. S8.** Detection of antibody to HA subtypes in bat sera by ELISA. Plates were coated with 1 ug/mL of H18 rHA antigen (homologous), or H17, H5,H1 rHA antigen (heterologous) to ascertain levels of cross reactivity. Serial log_2_ dilutions of sera were performed, starting at 1:500, and an absorbance reading was taken at 490 nm for 0.1 seconds. Samples represented are (A) Peru 017, (B) Peru 019, (C) Peru 020, and (D) Peru 031.
